# Myeloid *Mir34a* suppresses initiation and progression of intestinal and colitis-induced colon cancers in *APC*^*min*^ mice

**DOI:** 10.1038/s41419-026-08851-6

**Published:** 2026-05-15

**Authors:** Yun Chen, Fangteng Liu, Janine König, Nassim Bouznad, Heiko Hermeking

**Affiliations:** 1 Experimental and Molecular Pathology, Institute of Pathology, Ludwig-Maximilians-University München, Thalkirchnerstrasse 59, Munich, D-80337 Germany; 2 German Cancer Consortium (DKTK), Partner Site Munich, Munich, D-80336 Germany; 3 German Cancer Research Center (DKFZ), Heidelberg, D-69210 Germany

**Keywords:** Colon cancer, Experimental models of disease

## Abstract

Here we determined whether myeloid *Mir34a* has a tumor suppressive function in *Apc*^*Min/+*^ mice, a model for intestinal and colon cancer. Myeloid cell-specific deletion of *Mir34a* in *Apc*^*Min/+*^ mice increased tumor initiation and allowed progression towards invasive carcinomas, which are generally not observed in *Apc*^*Min/+*^ mice. Loss of *Mir34a* facilitated the polarization of tumor-associated macrophages (TAMs) towards a pro-tumorigenic M2-like state, implying that *Mir34a* is required to maintain TAMs in a tumor-suppressive state. Also, *Mir34a*-deficient, bone-marrow-derived macrophages (BMDMs) from *Apc*^*Min/+*^ mice were polarized towards a pro-tumorigenic, M2-like state and displayed enhanced migration when compared to *Mir34a*-proficient BMDMs. Intestinal tumors in myeloid *Mir34a*-deficient mice showed elevated expression of several known *Mir34a* target mRNAs, including *Csf1r, Pd-l1, Mmp9, Ccl22*, and *c-Myc*. In addition, the number of immuno-suppressive, pro-tumorigenic CD4^+^Foxp3^+^ T_reg_ cells increased in myeloid *Mir34a*-deficient intestinal tumors. Moreover, *Apc*^*Min/+*^ mice with myeloid-specific deletion of *Mir34a* had a significantly diminished survival rate. Following the induction of inflammatory colitis, these mice showed enhanced colon cancer initiation and progression towards invasive carcinomas with an increase in M2-like TAMs, N2-like neutrophils and T_reg_ cells. These findings imply that myeloid *Mir34a* suppresses tumor formation and progression by maintaining myeloid and T-cells in an anti-tumorigenic state. Therefore, the p53-*miR-34a* axis has a central role in non-tumor cell mediated suppression of intestinal and colon cancers.

## Introduction

The tumor microenvironment (TME) affects tumor initiation, progression and metastasis. In the recent years a complex interplay between cancer cells and diverse, non-malignant cell types present in the tumor micro-environment (TME) has been described: tumor-associated macrophages (TAMs), neutrophils (TANs) and myeloid-derived suppressor cells display immunosuppressive characteristics [1, 2], and have been associated with immunotherapy efficacy [3], as well as pre-metastatic niche formation [4]. TAMs and TANs have dual roles with both, tumor-promoting and tumor-restraining functions during tumor development, depending on their polarization state [5, 6]: M2-like TAMs and N2-like TANs facilitate tumor growth by promoting angiogenesis, metastasis and suppressing immune-responses towards the tumor. In contrast, M1-like TAMs and N1-like TANs exhibit pro-inflammatory and immune-stimulating activities, thereby enhancing an immune reaction against the tumor [7–9]. Therefore, selectively blocking or reprogramming TAMs and TANs in these states may represent an attractive therapeutic strategy [10].

The *p53* tumor suppressor gene encodes a transcription factor, which is activated by multiple extracellular and cellular stress signals. The *p53* gene is inactivated by mutations in approximately half of all human cancers [11–13] and in around 80% of advanced colorectal cancer (CRC) [14]. p53 has also been implicated in immunogenic processes and may be involved in antitumor immunity [15, 16].

The *miR-34a* gene is directly induced by p53 and encodes a microRNA with multiple tumor suppressive functions [17]. We have previously shown that intestinal-cell specific deletion of *Mir34a* in *Apc*^*Min/+*^ mice, which represent a model for early, MSS/micro-satellite stable CRC [18], enhances adenoma formation and progression with elevated immune cell infiltration [19].

Here, we determined that myeloid *Mir34a* suppresses neoplasia formation and progression in *Apc*^*Min/+*^ mice. Myeloid *Mir34a* restricted intestinal adenoma invasiveness through maintaining a tumor-suppressive polarization state of myeloid cells in the TME. Myeloid *Mir34a* also exerted tumor suppressive functions during DSS-induced colonic tumor development in *Apc*^*Min/+*^ mice. In both models, an increase of pro-tumorigenic, immunosuppressive T_reg_ cells was observed after deletion of myeloid *Mir34a*. In line with these in vivo observations, bone-marrow-derived macrophages/BMDMs isolated from mice with myeloid-specific *Mir34a* deletion displayed up-regulated M2-like markers, enhanced migration and increased expression levels of several known *Mir34a* target mRNAs. This study illuminates the tumor suppressive function of *Mir34a* in tumor-associated, myeloid cells in intestinal and colonic cancers.

## Materials And Methods

### Generation and handling of mice

To generate mice with myeloid *Mir34a*-deficiency (*Mir34a*^*ΔMye*^) *LysM-Cre* mice (B6.129P2-Lyz^tm1(cre)lfo/J^; purchased from Jackson Laboratories Stock: 004781) were crossed with *Mir34a*^*fl/fl*^ mice as described previously [20]. *Apc*^*Min/+*^ mice [21, 22] were obtained from Marlon Schneider at Ludwig-Maximilians-Universität, Munich, Germany. *Mir34a*^*ΔMye*^ mice were crossed with *Apc*^*Min/+*^ mice to obtain *Apc*^*Min/+*^*; Mir34a*^*ΔMye*^ mice. All mice were backcrossed on C57Bl/6 background for at least five generations. Co-housed *Apc*^*Min/+*^*; Mir34a*^*fl/fl*^ littermates were used as controls in all experiments. Mice at 1 month of age received 2% dextran sodium sulfate (DSS) in the drinking water for 7 days, followed by 29 days of regular water. At day 66, mice were sacrificed. For evaluation of micro-adenomas, 5-week-old mice were sacrificed. Overall survival rate of mice was calculated using the Kaplan-Meier method with GraphPad Prism 10. Mice were kept on a 12 h light/dark cycle in individually ventilated cages in specific pathogen-free facility at Institute of Pathology, Ludwig-Maximilians-University Munich, with chow (standard formulation) and water supply *ad libitum*. All animal protocols were approved by the local authorities (Regierung von Oberbayern, AZ:55.2-2532.Vet_02-21-111). Genotyping primers are listed in Table S1.

### Tissue preparation and tumor count

The entire intestinal tract was isolated, flushed with ice-cold PBS, and opened longitudinally. For 18-week-old, moribund, and DSS-treated *Apc*^*Min/+*^ mice, the small intestine was divided into three equal sections (duodenum, jejunum, ileum), while the colon was kept intact, each section was photographed, fixed as “Swiss-rolls” in formalin, dehydrated, and embedded in paraffin. Tumor numbers and area were evaluated using ImageJ.

### Aberrant crypt foci

Aberrant crypt foci (ACF) were stained as described elsewhere [23]. In brief, segments were fixed flat in 4% formalin for 24 h, stained with methylene blue (Merck) (0.2% in formalin) for 8–10 s and stored in 4% formalin for at least 24 h. The ACFs were scored under the light microscope at ×5 magnification.

### Histology and immunohistology

Two µm sections of paraffin-embedded tissues were stained using standard hematoxylin and eosin (H&E) protocols. Tumors were histologically classified as adenomas with low-, high-grade dysplasia or as invasive carcinomas. For Immunohistochemistry (IHC), sections were deparaffinized, rehydrated and boiled in antigen retrieval solution (Dako). After blocking, primary antibodies were applied and incubated at 4 °C overnight or 2 h room temperature (RT). Using DAB (3,3´-Diaminobenzidine) (Dako) the bound antibodies were visualized and counterstained with hematoxylin (Vector). Sections were scanned with a Vectra® Polaris™ Automated Quantitative Pathology imaging system and quantified using ImageJ. The kits and antibodies employed for this are listed in Tables S2 and S3.

### Indirect immunofluorescence analysis

Sections were deparaffinized, rehydrated, and boiled in antigen retrieval solution (Dako). After blocking with 5% goat-serum in PBS, the first primary antibody was applied overnight at 4 °C, followed by the second primary antibody for 2 h at RT. A mixture of two secondary antibodies was incubated subsequently for 1 h at RT in the dark. Nuclei were stained with DAPI (4’,6-Diamidino-2-Phenylindole, Dihydrochloride) (Roche, Switzerland) for 5 min in the dark at RT. Sections were scanned with a Vectra® Polaris™ Automated Quantitative Pathology imaging system and quantified using ImageJ software. Antibodies are listed in Table S3.

### Quantitative real-time PCR

RNA was extracted using RNeasy Mini Kit or miRNeasy Mini Kit (QIAGEN) and cDNA was synthesized using the Verso cDNA Kit (Thermo Scientific) or miRCURY LNA RT Kit (QIAGEN) following manufacturer’s instructions. A mix containing Fast SYBR Green Mastermix (Applied Biosystems or miRCURY LNA SYBR Green PCR Kit), dH2O, cDNA, and the indicated primers (listed in Table S1) was prepared, and samples were run on a Light Cycler 480 (Roche Diagnostics) using the program IDEAS 2.0. Relative gene expression levels were evaluated using the ΔΔCt method and normalized to *cyclophilin* expression. Kits are listed in Table S2.

### Cell culture

To obtain primary cells (BMDMs), femur and tibia of *Apc*^*Min/+*^ mice were collected, and bone marrow was flushed with ice-cold HBSS containing 10% FBS. The flush-through was filtered, centrifuged and cells were seeded in 10 mL RPMI medium containing 20% L929 conditioned medium. Twenty-four hours later, supernatant was collected, centrifuged, and re-seeded, followed by a change of medium to RPMI medium supplemented with 10% L929 conditioned medium on day 5 after isolation. Forty-eight hours later, cells were seeded. One day after seeding BMDMs were treated with 10 ng/mL murine IL-4 (Peprotech; #214-14), 100 ng/mL LPS (Sigma-Aldrich; #L4391) and 5 ng/mL murine IFN-γ (Peprotech; #315-05) for 4 h. The murine colorectal cancer cell line CT-26 was cultured in RPMI 1640 medium.

### Modified Boyden chamber assay

A total of 50,000 BMDMs per Transwell-insert were seeded in culture medium for 1 day into 24-well Transwell chambers (Greiner Bio One) with an 8 µM pore size. Two hours prior to the assay, the medium was changed to low FBS (0.5%). The lower chamber was filled with normal growth medium and the Transwell-inserts (upper chamber) with BMDMs were placed on top. After 4 and 24 h the Transwell-inserts were removed, BMDMs were fixed with 4% PFA, stained with 0.1% crystal violet and non-migratory cells on the top filter were removed. The cell number was quantified using ImageJ.

### Scratch assay

A total of 70 µL CT-26 cell suspension (1 × 10^6^ cells/mL) per insert-side (IBIDI, #80209) and 200,000 BMDMs were seeded per Transwell (Greiner Bio One). Two hours before co-cultivation 10 µg/mL Mitomycin C (Sigma-Aldrich; #M0503) was added to CT-26 cells. Followed by co-cultivation of CT-26 cells with *Mir34a*-proficient or *Mir34a*-deficient BMDMs obtained from *Apc*^*Min/+*^ mice for 24 h. The percentage of wound closure was measured with AxioVision SE (Zeiss).

### Statistical analysis

GraphPad Prism 10.1.0 was used to determine significant differences between groups via two-tailed unpaired Student’s *t* test. Values are represented as mean ± SEM. *p* values < 0.05 were regarded as statistically significant (*p < 0.05, **p < 0.01, ***p < 0.001, ****p < 0.0001).

## Results

### Myeloid *Mir34a* suppresses tumor formation and progression in *Apc*^*Min/+*^ mice

**Fig. 1:**
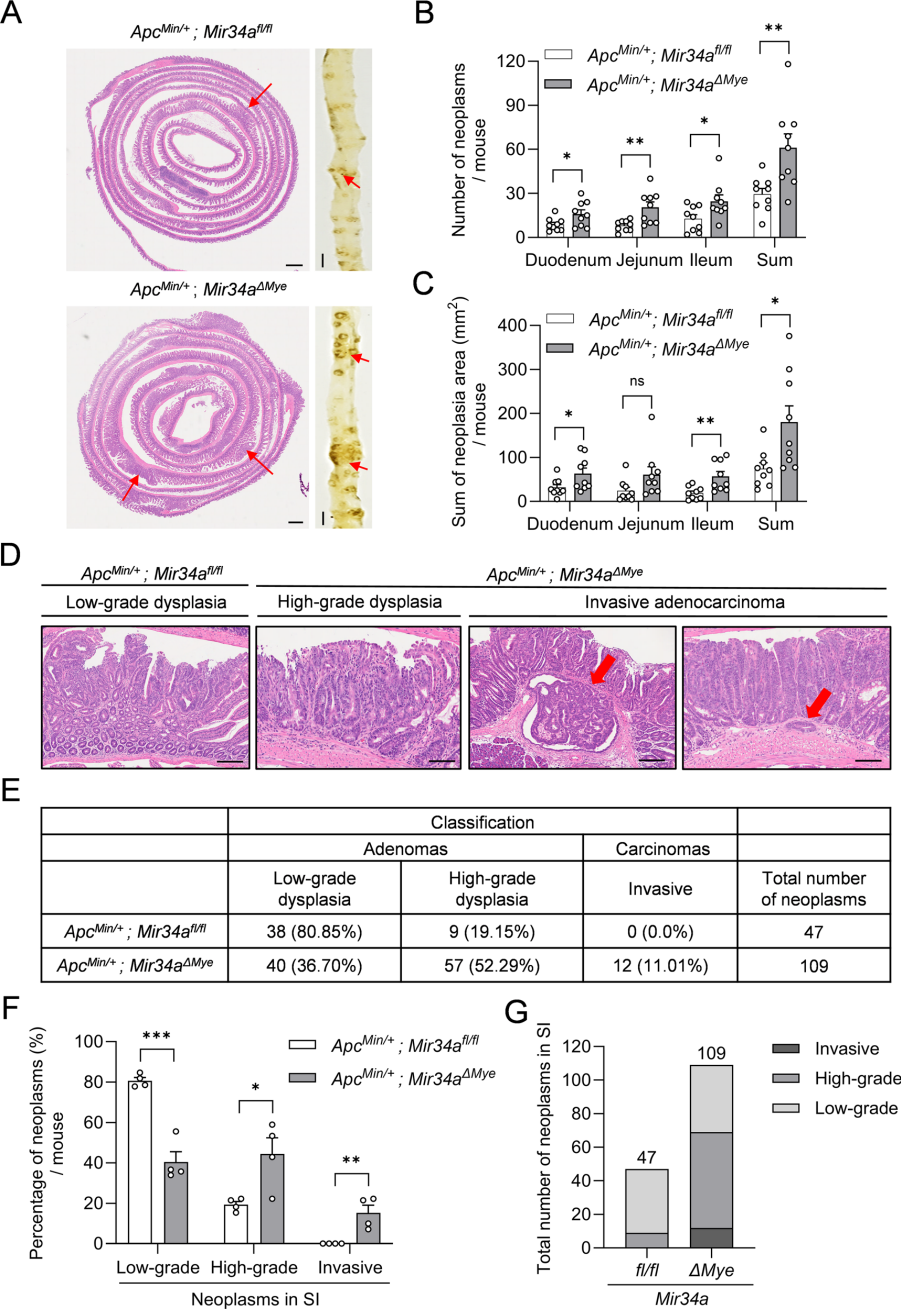
Myeloid *Mir34a* suppresses tumor formation and progression in *Apc*^*Min/+*^ mice. **A** Representative macroscopic images of polyps in resected small intestines of 18-week-old *Apc*^*Min/+*^ mice with the indicated genotypes. Scale bar: 2 cm. Representative H&E-stained “swiss-roll” sections of the small intestine of 18-week-old *Apc*^*Min/+*^ mice. Scale bar: 800 µm. **B** Quantification of the number of neoplasms within intestinal regions of 18-week-old *Apc*^*Min/+*^ mice with the indicated genotypes (*n* = 9 per genotype). **C** Sum of neoplasia area stratified by small intestinal region per mouse of *Apc*^*Min/+*^ mice with the indicated genotypes (*n* = 9 per genotype). **D** Representative pictures of different tumor stages in 18-week-old *Apc*^*Min/+*^ mice with the indicated genotypes. Scale bar: 200 µm. Red arrows correspond to invasive adenocarcinomas. **E**–**G** Quantification of different tumor stages and tumor number in neoplasms from the small intestine in 18-week-old *Apc*^*Min/+*^ mice with the indicated genotypes (*n* = 4 per genotype). SI small intestine. **B**, **C**, **F** Mean values ± SEM are provided. Student’s *t* test was used to determine significance. *p < 0.05, **p < 0.01, ***p < 0.001, ns, not significant.

To determine whether myeloid *Mir34a* affects intestinal tumor formation, we crossed *LysM-Cre/Mir34a*^*fl/fl*^ mice with adenoma-prone *Apc*^*Min/+*^ mice on a C57BL/6 background to generate *Apc*^*Min/+*^ mice with deletion of *Mir34a* specifically in myeloid cells (*Apc*^*Min/+*^*; Mir34a*^*ΔMye*^). At the age of 18 weeks, these mice displayed a more than 2-fold increase in the total number of intestinal neoplasms when compared to *Apc*^*Min/+*^ control mice (Fig. 1A–C). This effect was not observed in the colon (Fig. S1A–C). In addition, the number of high-grade adenomas was increased in age-matched *Apc*^*Min/+*^*; Mir34a*^*ΔMye*^ mice when compared to control mice (Fig. 1D–G). While neoplasms in *Mir34a*-proficient *Apc*^*Min/+*^ mice were predominantly low-grade adenomas and not invasive, in myeloid *Mir34a*-deficient mice, approximately 52% of neoplasms progressed to high-grade adenomas and 11% developed into invasive carcinomas (Fig. 1E–G). We also observed that, in age-matched, 18-week-old *Apc*^*Min/+*^*; Mir34a*^*ΔMye*^ mice, the total length of the small intestine, as well as the width and depth of the crypts, were significantly increased (Fig. S1D–F). In contrast, the total length and architecture of crypts in the colon were similar in both genotypes (Fig. S1G–I). However, as the increase in length was in the range of 9%, it could not account for the increased number of neoplasms observed in the myeloid *Mir34a*-deficient mice. Taken together, *Mir34a*-deficiency in the myeloid lineage enhanced tumor formation and progression in the small intestine of *Apc*^*Min/+*^ mice, suggesting that a physiological function of myeloid *Mir34a* is to suppress intestinal tumor initiation and progression.

### Myeloid *Mir34a* modulates intestinal TME in *Apc*^*Min/+*^ mice

**Fig. 2:**
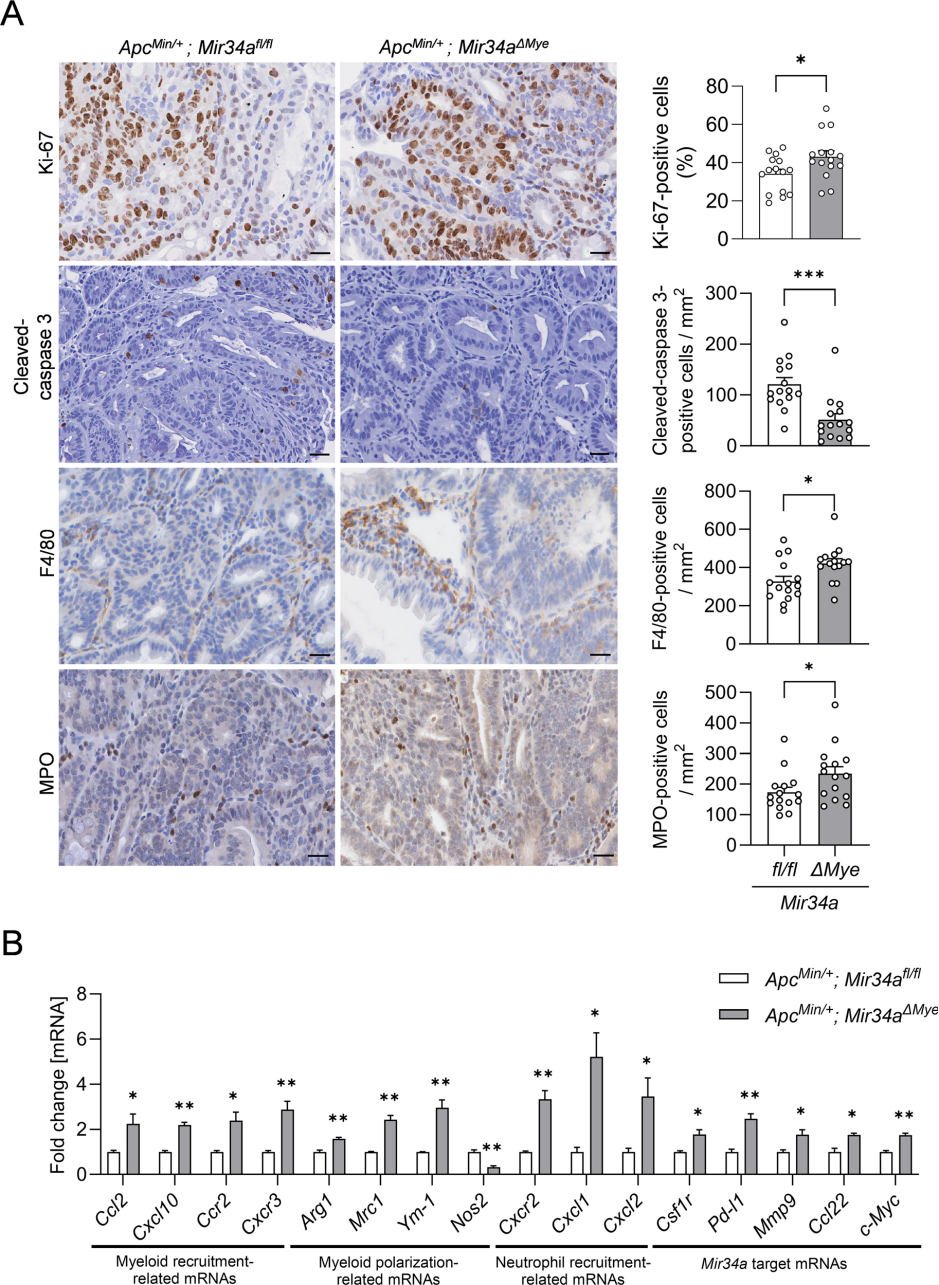
Myeloid *Mir34a* modulates intestinal TME in *Apc*^*Min/+*^ mice. **A** Immunohistochemical detection of Ki-67, cleaved-caspase 3, F4/80 and Myeloperoxidase (MPO) in neoplasms from the small intestine in 18-weeks-old *Apc*^*Min/+*^ mice with the indicated genotypes (left panel). Scale bars: 40 µm. Quantification of Ki-67^+^, cleaved-caspase 3^+^, F4/80^+^ and MPO^+^ cells (right panel). **B** QPCR detection of the indicated mRNAs in neoplasms from the small intestine in 18-week-old *Apc*^*Min/+*^ mice with the indicated genotypes. **A**, **B** Three mice per genotype were evaluated. Mean values ± SEM are provided. Student’s *t* test was used to determine significance. *p < 0.05, **p < 0.01, ***p < 0.001.

Next, we determined whether loss of myeloid *Mir34a* affects cellular properties within intestinal tumors and the distribution of cell types in the TME of *Apc*^*Min/+*^ mice. Indeed, an increase of Ki-67-positive cells and a lower number of cleaved-caspase 3-positive tumor cells in intestinal and colonic neoplasms was observed in 18-week-old *Apc*^*Min/+*^ mice with myeloid *Mir34a*-deficiency when compared to their *Apc*^*Min/+*^ control littermates (Figs. 2A and S2A). Therefore, loss of myeloid *Mir34a* increases proliferation and decreases apoptosis of tumor cells. Furthermore, a significant increase in MPO^+^ neutrophils and F4/80^+^ macrophages in tumors and in their vicinity was detected in myeloid *Mir34a-*deficient *Apc*^*Min/+*^ mice (Fig. 2A). Therefore, myeloid *Mir34a* may suppress the accumulation of macrophages and neutrophils in the TME of intestinal tumors.

Next, we investigated whether myeloid *Mir34a* might influence the expression of molecules involved in the recruitment and polarization of myeloid cells. Indeed, intestinal neoplasms showed an up-regulation of mRNAs encoding myeloid cell recruitment-related cytokines (*Ccr2, Ccl2, Cxcl10* and *Cxcr3*), as well as neutrophil recruitment-associated cytokines (*Cxcr2, Cxcl1* and *Cxcl2*) in 18-week-old *Apc*^*Min/+*^*; Mir34a*^*ΔMye*^ mice when compared to control mice (Fig. 2B). These findings indicate that cytokines, which exert myeloid cell-recruitment functions, are secreted to a higher extent in neoplasms with *Mir34a*-deficient myeloid cells.

Additionally, the expression of genes associated with tumor-promoting M2-like TAMs, such as *Arg1*, *Mrc1* and *Ym-1* mRNAs, was elevated in neoplasms from *Apc*^*Min/+*^*; Mir34a*^*ΔMye*^ mice, whereas the expression of the marker *Nos2*, characteristic for anti-tumorigenic M1-like TAMs, was reduced when compared with neoplasms from age-matched *APC*^*Min/+*^ ; *Mir34a*^*fl/fl*^ mice (Fig. 2B). These results imply that myeloid *Mir34a* maintains intra-tumoral TAMs in an M1-like state through regulating the expression of polarization-related cytokines.

Furthermore, we detected an increased expression of several known *Mir34a* target mRNAs, such as *Csf1r, Pd-l1, Mmp9, Ccl22*, and *c-Myc*, in neoplasms of *Apc*^*Min/+*^*; Mir34a*^*ΔMye*^ mice (Fig. 2B). The concomitant increase of these *Mir34a* targets, including invasion-related factors, such as *Mmp9* and *c-Myc*, the macrophage polarization-related factor, *Csf1r*, and immune-regulatory factors, such as *Pd-l1*, and *Ccl22*, might play an important role in driving the enhanced invasiveness of adenomas observed in *Apc*^*Min/+*^*; Mir34a*^*ΔMye*^ mice. Taken together, these findings suggest that myeloid *Mir34a* is critical for maintaining a tumor-suppressive state of myeloid cells within the TME of *Apc*^*Min/+*^ mice.

### *Mir34a* affects myeloid polarization and T-cell recruitment in 18-week-old *Apc*^*Min/+*^ mice

**Fig. 3:**
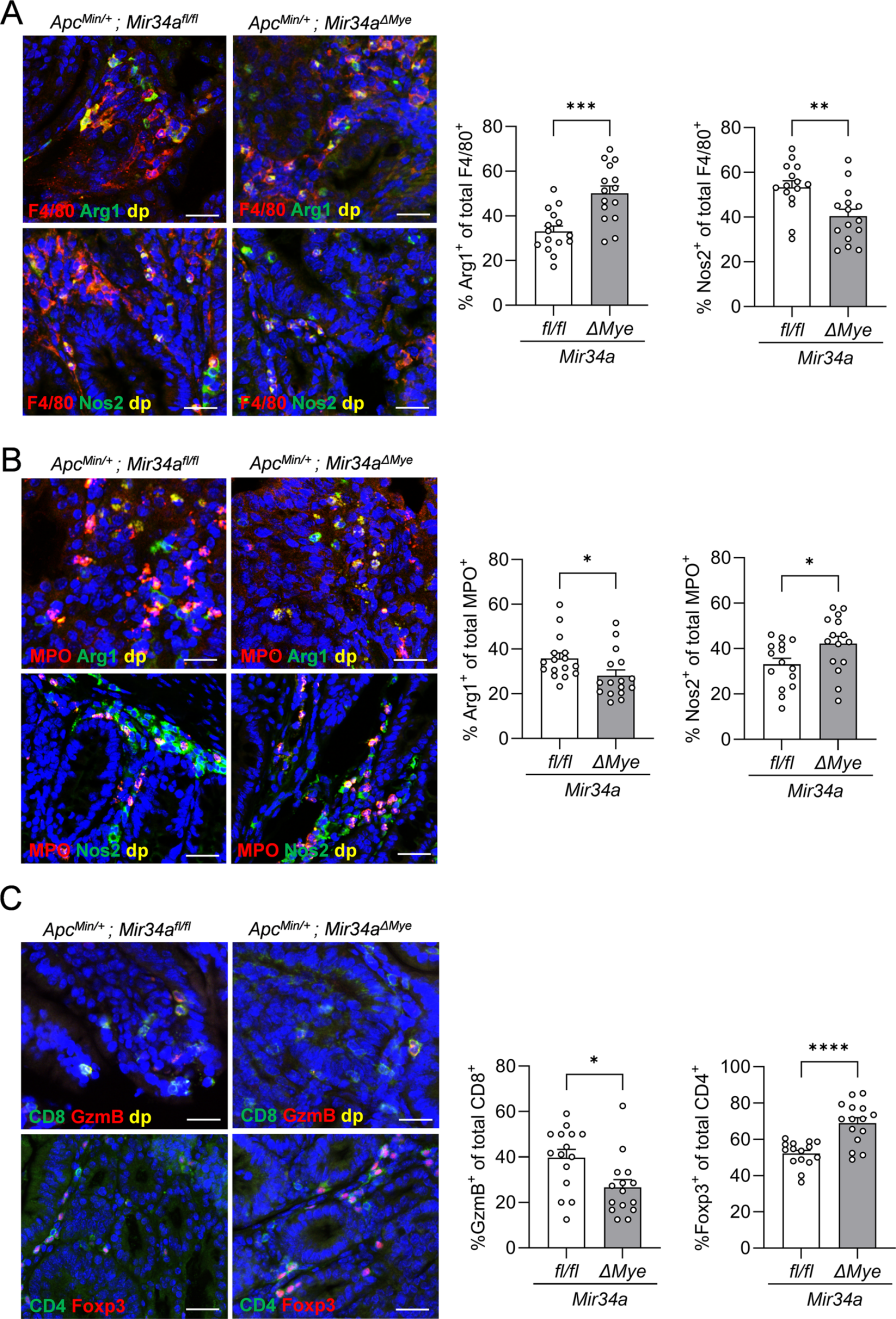
*Mir34a* affects myeloid polarization and T-cell recruitment in 18-week-old *Apc*^*Min/+*^ mice. **A** Representative pictures of macrophages in the small intestine of 18-week-old *Apc*^*Min/+*^ mice with the indicated genotypes. F4/80 (red), Arg1 (green), Nos2 (green), DAPI (blue), and dp (double positive, yellow). Scale bar: 20 µm. Quantification of F4/80^+^Arg1^+^ and F4/80^+^Nos2^+^ cells. **B** Representative pictures of neutrophils in the small intestine of 18-week-old *Apc*^*Min/+*^ mice with the indicated genotypes. MPO (red), Arg1 (green), Nos2 (green), DAPI (blue), and dp (double positive, yellow). Scale bar: 20 µm. Quantification of MPO^+^Arg1^+^ and MPO^+^Nos2^+^ cells. **C** Representative pictures of Granzyme-B-positive cytotoxic T-cells and Foxp3-positive regulatory T-cells in the small intestine of 18-week-old *Apc*^*Min/+*^ mice with the indicated genotypes. Granzyme-B (red), CD8 (green), Foxp3 (red), CD4 (green), DAPI (blue), and dp (double positive, yellow). Scale bar: 20 µm. Quantification of CD8^+^GzmB^+^ and CD4^+^Foxp3^+^ cells. **A**–**C** Three mice per genotype were evaluated. Mean values ± SEM are provided. Student’s *t* test was used to determine significance. *p < 0.05, **p < 0.01, ***p < 0.001, ****p < 0.0001.

Arginase1 (Arg1) and nitric oxide synthase 2 (Nos2) are known markers for pro-tumorigenic, M2/N2-like (Arg1) and anti-tumorigenic, M1/N1-like (Nos2) polarization states of TAMs and TANs [24–26]. In order to determine the polarization state of TAMs and TANs, we next utilized indirect immunofluorescence detection to evaluate the frequency of Arg1^+^ and Nos2^+^ myeloid cells within neoplasms. Co-immunostaining of intestinal neoplasms revealed a significant increase by approximately 20% in the number of F4/80^+^Arg1^+^ cells and a significant decrease by about 13% in the number of F4/80^+^Nos2^+^ cells in age-matched *Apc*^*Min/+*^*; Mir34a*^*ΔMye*^ mice (Figs. 3A and S3A, B). Similar results were obtained by immunohistochemical detection of single markers (Fig. S2B). Surprisingly, we observed an increase in Nos2-positive MPO^+^ neutrophils in the tumor tissue of myeloid *Mir34a-*deficient mice, whereas Arg1-positive MPO^+^ cells were decreased (Figs. 3B and S3C, D).

Next, we investigated whether the increase in M2-like TAMs due to myeloid *Mir34a-*deficiency affects T-cell infiltration and composition in 18-week-old *Apc*^*Min/+*^ mice. Quantification of CD3^+^CD8^+^ T-cells showed a significant decrease in 18-week-old *Apc*^*Min/+*^*; Mir34a*^*ΔMye*^ mice when compared to control mice (Fig. S4A). On the contrary, we observed an increase of CD3^+^CD4^+^ T-cells in the tumors of myeloid *Mir34a*-deficient mice (Fig. S4B). To further characterize the functional state of these T-cell populations, we next evaluated tumors for cytotoxic CD8^+^ T-cells and regulatory T-cells (T_regs_). An increase of CD4^+^Foxp3^+^T-cells ( = T_regs_) was observed in adenomas of myeloid *Mir34a-*deficient mice, whereas Granzyme-B-positive CD8^+^ T-cells were significantly decreased (Figs. 3C and S4C, D). Therefore, myeloid *Mir34a*-deficiency skews the T-cell compartment towards an immunosuppressive, pro-tumorigenic state.

Overall, myeloid-specific loss of *Mir34a* leads to a shift towards anti-inflammatory, tumor-supportive M2-like macrophages and immune-suppressive CD4^+^Foxp3^+^ T_reg_ cells, while simultaneously driving the polarization of pro-inflammatory N1-like neutrophils. Therefore, myeloid *Mir34a* suppresses intestinal tumor development of *Apc*^*Min/+*^ mice by maintaining the polarization of TAMs and TANs in an anti-tumorigenic state, which also affects tumor-associated T-cells, such as T_regs_.

### *Mir34a* regulates polarization in BMDMs

**Fig. 4:**
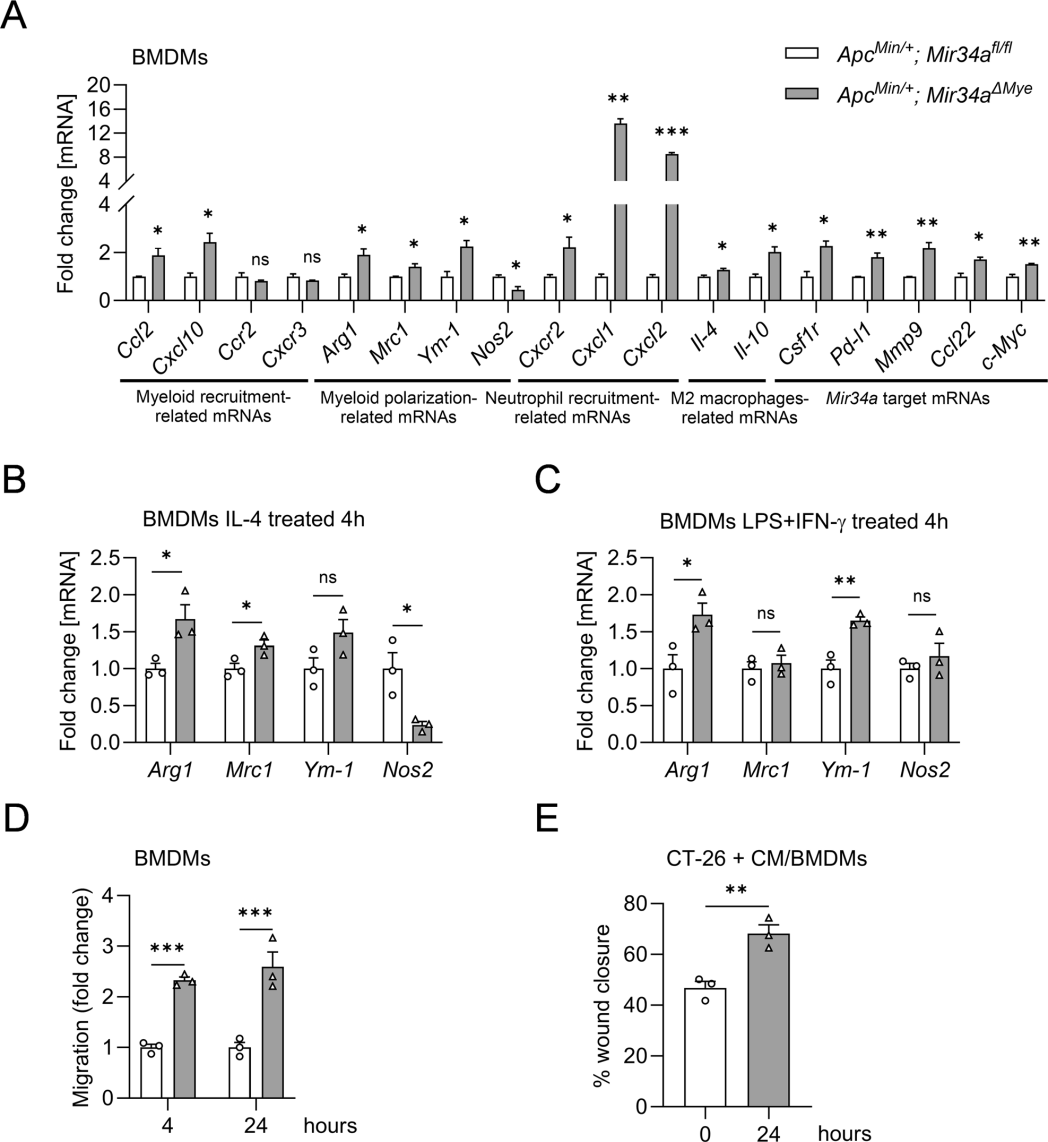
*Mir34a* regulates polarization in BMDMs. **A** QPCR detection of the expression of the indicated *Mir34a* target mRNAs in BMDMs of *Apc*^*Min/+*^ mice with the indicated genotypes. **B** Expression of polarization-associated genes in BMDMs after IL-4 treatment for 4 h in *Apc*^*Min/+*^ mice with the indicated genotypes. **C** Expression of polarization-associated genes in BMDMs after LPS + IFN-γ treatment for 4 h in *Apc*^*Min/+*^ mice with the indicated genotypes. **D** Migration of BMDMs derived from the indicated *Apc*^*Min/+*^ mice after 4 and 24 h. **E** Percentage of wound closure after 24 h co-culture of BMDMs and CT26 cells. CM, conditioned media. **A**–**E** Three mice per genotype were evaluated. Mean values ± SEM are provided. Student’s *t* test was used to determine significance. *p < 0.05, **p < 0.01, ***p < 0.001, ns, not significant.

In order to analyze the function of myeloid *Mir34a* on the cellular level, we next isolated BMDMs from *Apc*^*Min/+*^*; Mir34a*^*fl/fl*^ and *Apc*^*Min/+*^*; Mir34a*^*ΔMye*^ mice. The deletion of *Mir34a* was confirmed on the level of *pri-Mir34a* and mature Mir34a expression in BMDMs (Fig. S5A). *Mir34a*-deficient BMDMs displayed an up-regulation of myeloid-specific recruitment and differentiation markers, which confirms the observations made in mice deficient for myeloid *Mir34a* (Fig. 4A). These results indicate that myeloid *Mir34a* regulates myeloid recruitment and differentiation. Also, a significant increase of tumor-promoting/myeloid cell polarization related genes, *Arg1*, *Ym-1* and *Mrc1*, as well as a significant decrease of the tumor-restricting/myeloid cell polarization related *Nos2* mRNA was observed, when compared to myeloid *Mir34a*-proficient BMDMs (Fig. 4A). In addition, up-regulation of M2-like macrophage-related mRNAs, such as *Il-4* and *Il-10*, as well as several known *Mir34a* targets (*Csf1r, Pd-l1, Mmp9, Ccl22, and c-Myc*), was detected in *Mir34a*-deficient BMDMs, when compared to myeloid *Mir34a*-proficient BMDMs (Fig. 4A).

Next, we polarized BMDMs to an M1-like state by exposure to LPS and IFN-γ, as well as to an M2-like state by IL-4 treatment. IL-4 led to elevated levels of *Arg1*, *Mrc1*, and *Ym-1* in *Mir34a*-deficient BMDMs when compared to *Mir34a*-proficient BMDMs (Figs. 4B and S5B–D). Therefore, myeloid *Mir34a*-deficiency augments pro-tumoral M2-like polarization in BMDMs, which is consistent with the in vivo effects of myeloid *Mir34a-*deficiency observed above. After treatment with LPS and IFN-γ, *Mir34a*-deficient BMDMs maintained elevated expression of *Arg1* and *Ym-1*, while Nos2 showed a non-significant increase compared with *Mir34a*-proficient BMDMs (Figs. 4C and S5E). These observations imply that *Mir34a* functions to maintain an M1-like polarization and thereby suppresses tumor progression.

Furthermore, a modified Boyden chamber assay showed that BMDMs with *Mir34a*-deficiency harbor enhanced migration capabilities when compared to control BMDMs (Figs. 4D and S5F). In order to determine whether myeloid *Mir34a* may affect tumor cell migration, we co-cultured murine CT-26 CRC cells with *Mir34a*-proficient or *Mir34a*-deficient BMDMs isolated from *Apc*^*Min/+*^ mice (Figs. 4E and S5G). CT-26 cells co-cultured with myeloid *Mir34a-*deficient BMDMs closed a scratch wound more efficiently than the controls, implying that myeloid *Mir34a* may prevent CRC migration by regulating the secretion of factors that influence the migration of neighboring CRC cells. Taken together, the findings obtained by analyzing *Mir34a*-deficient BMDMs further support the conclusion, that myeloid *Mir34a* suppresses tumor progression by restricting the shift of TAMs towards a tumor-promoting M2-like phenotype, which enhances migration and presumably other tumorigenic features of CRC cells, such as invasion.

### Myeloid *Mir34a* deletion decreases the survival time of *Apc*^*Min/+*^ mice

**Fig. 5:**
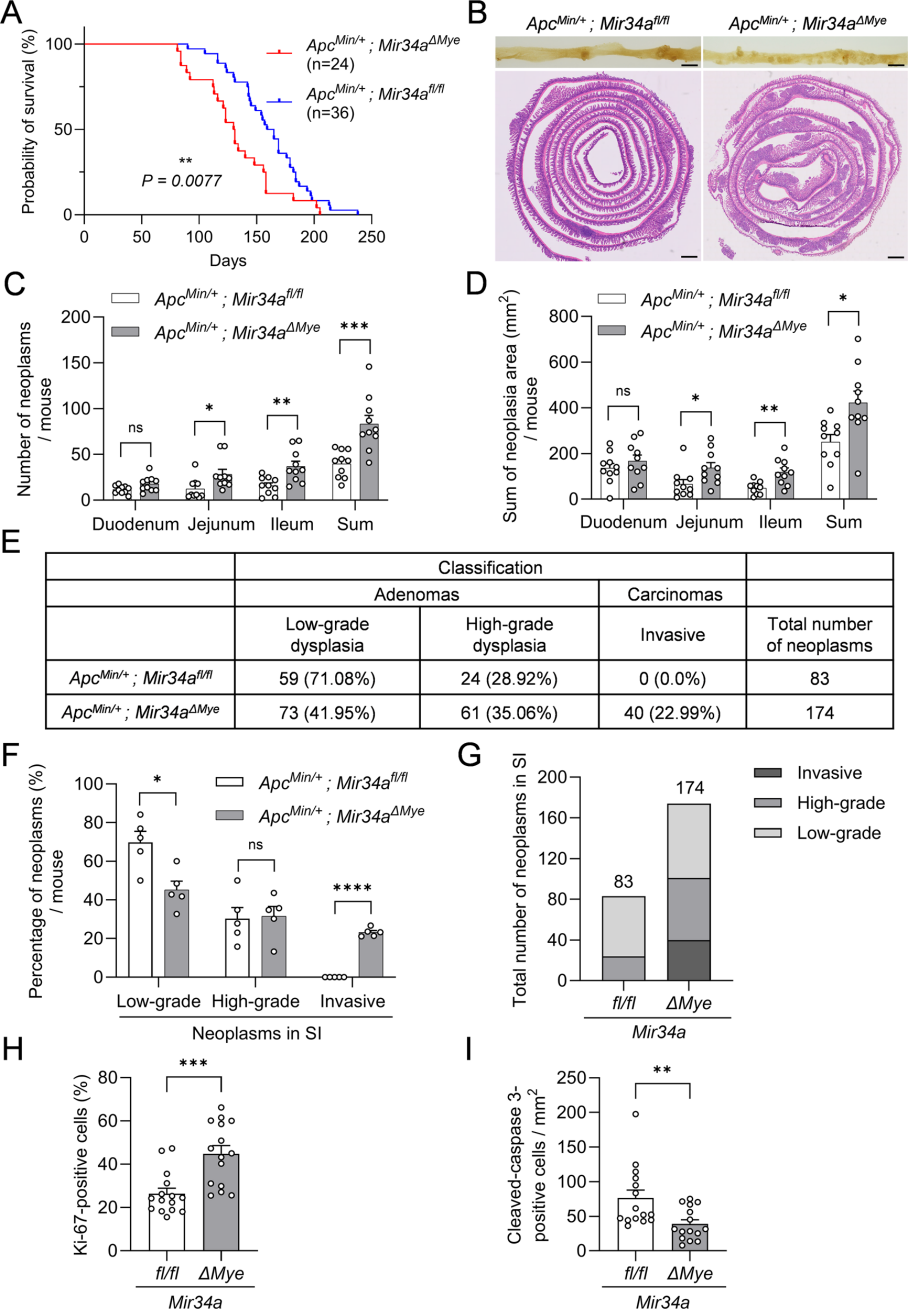
Myeloid *Mir34a* deletion decreases survival time of *Apc*^*Min/+*^ mice. **A** Kaplan-Meier survival analysis of *Apc*^*Min/+*^ mice with the indicated genotypes. Results were compared with a log-rank test. **B** Representative macroscopic images of polyps in resected small intestines of moribund *Apc*^*Min/+*^ mice with the indicated genotypes (upper panel, Scale bar: 1 cm). Representative H&E stained “swiss-roll” sections of the small intestine of moribund *Apc*^*Min/+*^ mice (lower panel, Scale bar: 800 µm). **C** Quantification of the number of neoplasms stratified by small intestinal region per mouse of moribund *Apc*^*Min/+*^ mice with the indicated genotypes (*n* = 10 per genotype). **D** Sum of neoplasia area stratified by small intestinal region per mouse of *Apc*^*Min/+*^ mice with the indicated genotypes (*n* = 10 per genotype). Quantification of **E** tumor stage and **F**, **G** tumor number from the small intestine in moribund *Apc*^*Min/+*^ mice with the indicated genotypes (*n* = 5 per genotype). SI small intestine. Quantification of immunohistochemistry of **H** Ki-67 and **I** cleaved-caspase 3 in neoplasms from the small intestine in moribund *Apc*^*Min/+*^ mice with the indicated genotypes (*n* = 3 per genotype). **C**, **D**, **F**, **H**, **I** Mean values ± SEM are provided. Student’s *t* test was used to determine significance. *p < 0.05, **p < 0.01, ***p < 0.001, ****p < 0.0001, ns, not significant.

Next, we determined whether myeloid *Mir34a* also influences the overall survival of *Apc*^*Min/+*^ mice. Indeed, *Apc*^*Min/+*^*; Mir34a*^*ΔMye*^ mice showed a shortened survival time with a median of 130 days, compared to 162 days in *Apc*^*Min/+*^ control mice (Fig. 5A). A similar shorter overall survival was observed in both male and female mice with myeloid *Mir34a*-deficiency (Fig. S6A), indicating that these effects of myeloid *Mir34a* do not depend on gender. In the moribund mice deletion of *Mir34a* in the myeloid lineage resulted in a 2-fold increase of the total number of neoplasms (Fig. 5B, C). Moreover, an increased frequency of tumors was found in all regions of the small intestine in *Apc*^*Min/+*^*; Mir34a*^*ΔMye*^ mice (Fig. 5D). Furthermore, the invasive carcinomas were only detected in the small intestine of *Apc*^*Min/+*^*; Mir34a*^*ΔMye*^ mice when compared to *Apc*^*Min/+*^*; Mir34a*^*fl/fl*^ mice (Figs. 5E–G and S6B). Therefore, myeloid *Mir34a* presumably suppresses progression of adenomas to invasive carcinomas. These differences only reached significance in the small intestine but not in the colon (Fig. S6C–E). In addition, an increase in Ki-67-positive cells and a decrease in apoptotic cells was detected in neoplasms from *Apc*^*Min/+*^*; Mir34a*^*ΔMye*^ mice (Figs. 5H, I and S6F), which might be responsible for the increase in the number and size of neoplasms. Altogether, these findings demonstrate that myeloid *Mir34a* inhibits tumor growth, delays adenoma progression and thereby presumably enhances overall survival in *Apc*^*Min/+*^ mice.

### Myeloid *Mir34a* suppresses early adenoma formation in 5-week-old *Apc*^*Min/+*^ mice

**Fig. 6:**
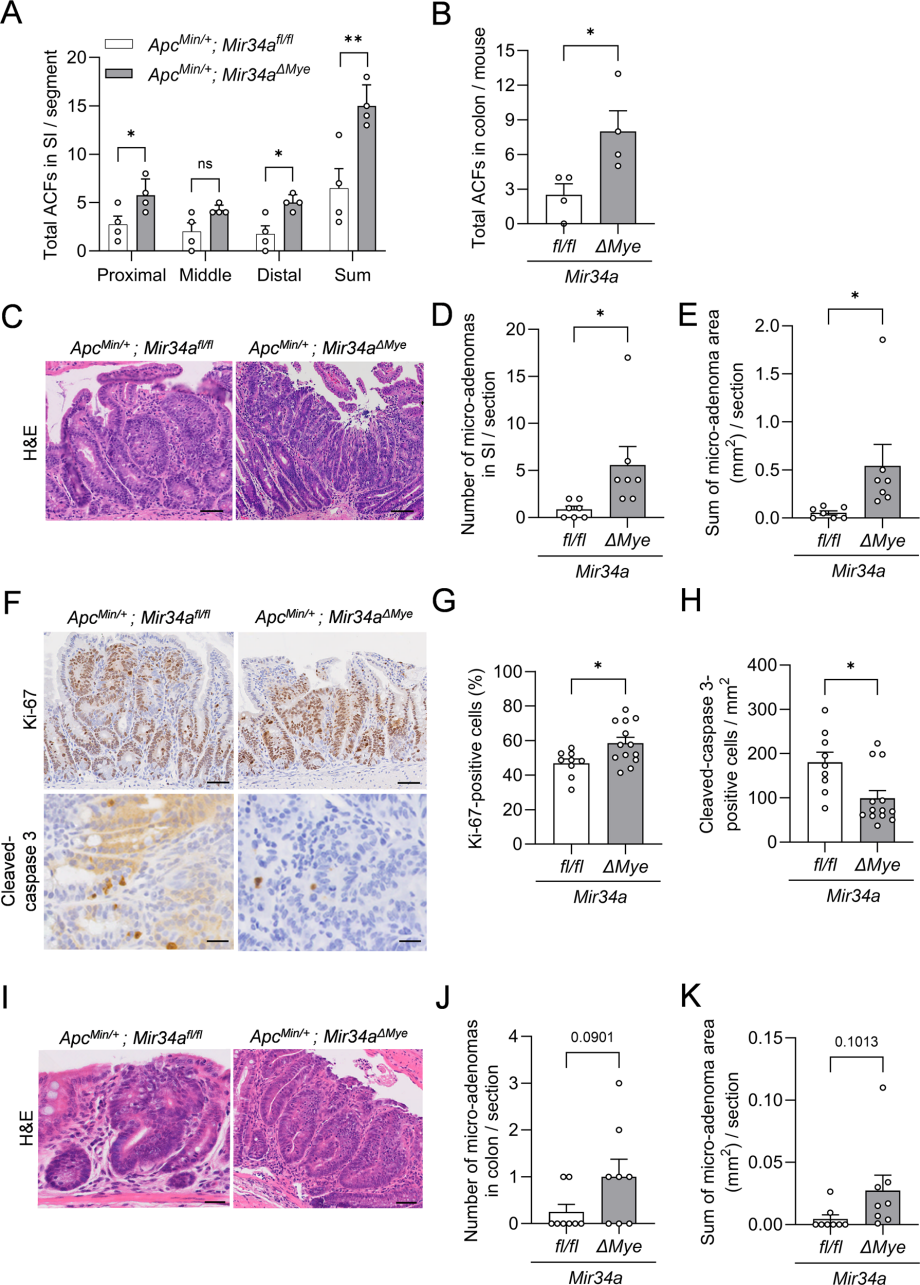
Effect of myeloid *Mir34a* on early adenoma formation in 5-week-old *Apc*^*Min/+*^ mice. **A** Quantification of the number of ACFs per segment of small intestine in 5-week-old *Apc*^*Min/+*^ mice with the indicated genotypes (*n* = 4 per genotype). SI small intestine, ACF aberrant crypt foci. **B** Quantification of the number of ACFs in total colon in 5-week-old *Apc*^*Min/+*^ mice with the indicated genotypes (*n* = 4 per genotype). **C** Representative H&E stainings of micro-adenoma in small intestine in 5-week-old *Apc*^*Min/+*^ mice with the indicated genotypes. Scale bar: 100 µm. **D** Quantification of the number of micro-adenomas in the small intestine per mouse of 5-week-old *Apc*^*Min/+*^ mice with the indicated genotypes (*n* = 7 per genotype). **E** Sum of micro-adenoma area per mouse in the small intestine of 5-week-old *Apc*^*Min/+*^ mice with the indicated genotypes (*n* = 7 per genotype). **F** Representative pictures of Ki-67- and cleaved-caspase 3-positive stained cells in micro-adenomas from the small intestine of 5-week-old *Apc*^*Min/+*^ mice with the indicated genotypes. Scale bars: 50 µm. Quantification of **G** Ki-67 and **H** cleaved-caspase 3 in micro-adenomas from the small intestine in 5-week-old *Apc*^*Min/+*^ mice with the indicated genotypes (*n* = 3 per genotype). **I** Representative H&E staining pictures of micro-adenoma in colon of 5-week-old *Apc*^*Min/+*^ mice with the indicated genotypes. Scale bar: 50 µm. **J** Quantification of the number of micro-adenomas in colon per mouse of 5-week-old *Apc*^*Min/+*^ mice with the indicated genotypes (*n* = 8 per genotype). **K** Sum of micro-adenoma area per mouse in colon of 5-week-old *Apc*^*Min/+*^ mice with the indicated genotypes (*n* = 8 per genotype). **A**, **B**, **D**, **E**, **G**, **H**, **J**, **K** Mean values ± SEM are provided. Student’s *t* test was used to determine significance. *p < 0.05, **p < 0.01, ns, not significant.

To investigate the effect of myeloid *Mir34a* on early-stage tumorigenesis in *Apc*^*Min/+*^ mice, we examined the number of aberrant crypt foci (ACF), which are considered to represent the initial stage of intestinal tumor development [27]: 5-week-old *Apc*^*Min/+*^*; Mir34a*^*ΔMye*^ mice exhibited a significantly elevated number of ACFs in both small intestine and colon (Figs. 6A, B and S7A). Moreover, we quantified early neoplastic transformation in *Apc*^*Min/+*^ mice using β-catenin and H&E staining: myeloid *Mir34a-*deficient *Apc*^*Min/+*^ mice showed an elevated rate of dysplasia initiation in SI when compared with control *Apc*^*Min/+*^ mice (Figs. 6C, D and S7B, C). Furthermore, the microadenoma area was increased in the small intestine of 5-week-old *Apc*^*Min/+*^*; Mir34a*^*ΔMye*^ mice when compared to their control littermates (Fig. 6E) presumably due to an increase in proliferation and a decrease in apoptosis (Fig. 6F–H). Additionally, the number and area of colonic micro-adenomas was also increased in 5-week-old *Apc*^*Min/+*^*; Mir34a*^*ΔMye*^ mice (Fig. 6I–K). In summary, our results indicate that myeloid *Mir34a* suppresses tumor initiation in the small intestine and colon of *Apc*^*Min/+*^ mice.

### Myeloid *Mir34a* suppresses colon cancers in DSS-treated *Apc*^*Min/+*^ mice

**Fig. 7:**
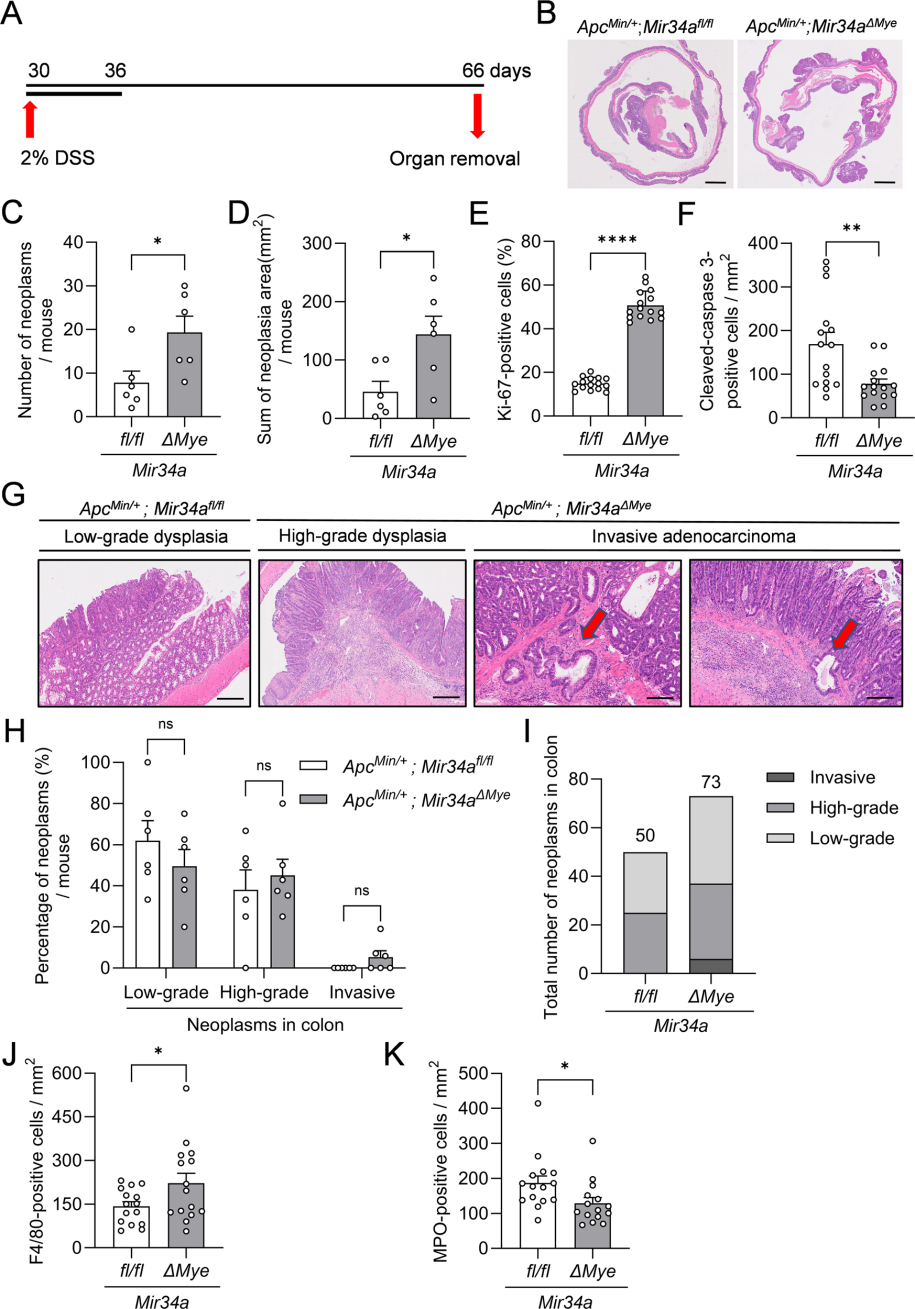
Myeloid *Mir34a* suppresses colon cancers in DSS-treated *Apc*^*Min/+*^ mice. **A** Schematic illustration of the experimental design. **B** Representative H&E stained “Swiss-roll” sections of 2% DSS treated *Apc*^*Min/+*^ mice. Scale bar: 2 mm. **C** Quantification of the number of neoplasms in the colon of 2% DSS treated *Apc*^*Min/+*^ mice with the indicated genotypes (*n* = 6 per genotype). **D** Sum of neoplasia area in the colon of 2% DSS treated *Apc*^*Min/+*^ mice with the indicated genotypes (*n* = 6 per genotype). Quantification of **E** Ki-67^+^ and **F** cleaved-caspase 3^+^ cells in neoplasms in 2% DSS treated *Apc*^*Min/+*^ mice with the indicated genotypes (*n* = 3 per genotype). **G** Representative pictures of different tumor stages in 2% DSS treated *Apc*^*Min/+*^ mice with the indicated genotypes. Scale bar: 100 µm. Red arrows correspond to invasive adenocarcinomas. **H**, **I** Quantification of different tumor stages and tumor number in neoplasms from colon in 2% DSS treated *Apc*^*Min/+*^ mice with the indicated genotypes (*n* = 6 per genotype). Quantification of **J** F4/80^+^ and **K** MPO^+^ cells in neoplasms from colon in 2% DSS treated *Apc*^*Min/+*^ mice with the indicated genotypes (*n* = 3 per genotype). **C**–**F**, **H**, **J**, **K** Mean values ± SEM are provided. Student’s *t* test was used to determine significance. *p < 0.05, **p < 0.01, ****p < 0.0001, ns, not significant.

Next, we investigated the effect of myeloid *Mir34a* loss on colorectal cancer formation in *Apc*^*Min/+*^ mice, which can be induced by treatment with dextran sodium sulfate (DSS) [28]. We evaluated the colon-carcinoma burden of mice following treatment with 2% DSS for a 7-day cycle (Fig. 7A). *Apc*^*Min/+*^*; Mir34a*^*ΔMye*^ mice demonstrated a significant increase in both, number and size of colon cancers, as well as a rise in the proportion of Ki-67-positive cells (Figs. 7B–F and S8A). A concomitant decrease of cleaved-caspase 3-positive cells was observed, when compared to control mice. These findings indicate that the enhanced number and size of colon cancers in myeloid *Mir34a*-deficient *Apc*^*Min/+*^ mice is presumably driven by higher proliferation rates and less apoptosis. Additionally, mice with myeloid *Mir34a*-deficiency showed an increased number of high-grade adenomas and invasive adenocarcinomas, whereas the number of low-grade adenomas was decreased when compared to control littermates. Notably, invasive adenocarcinomas were only observed in myeloid *Mir34a*-deficient mice, indicative of a suppressive effect of myeloid *Mir34a* on cancer cell invasiveness (Figs. 7G–I and S8B).

Collectively, these results showed that myeloid *Mir34a*-deficient mice are more susceptible to the formation of DSS-induced colon cancer, indicating a protective role of myeloid *Mir34a* against DSS-induced colon carcinomas in *Apc*^*Min/+*^ mice.

### *Mir34a* influences myeloid polarization and T-cell recruitment in DSS-treated *Apc*^*Min/+*^ mice

**Fig. 8:**
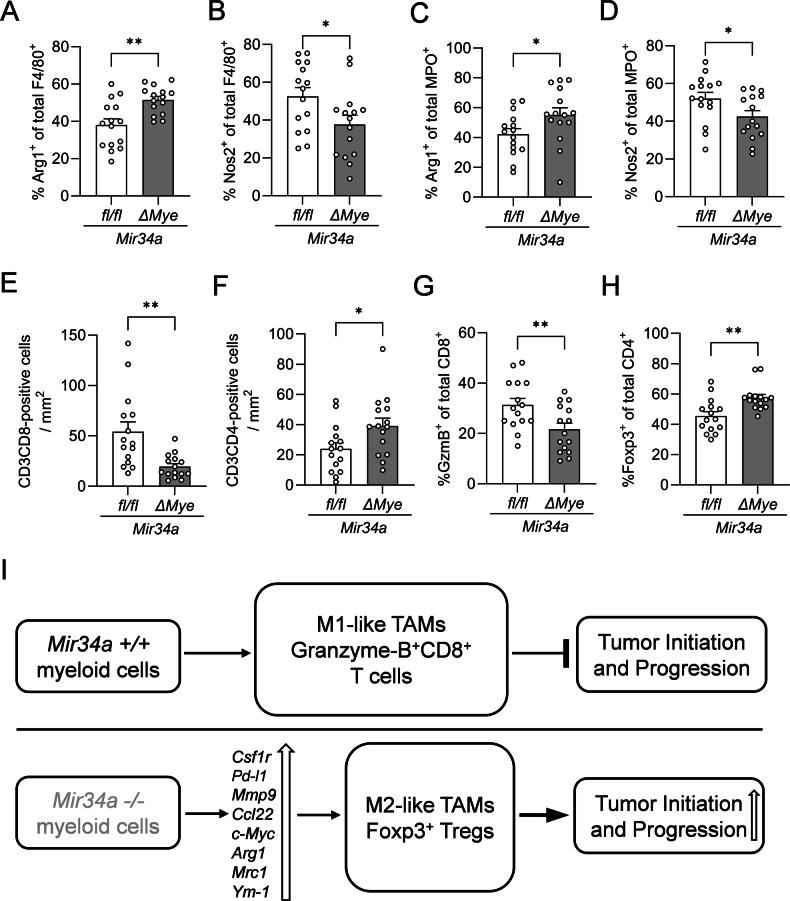
*Mir34a* influences myeloid polarization and T-cell recruitment in DSS-treated *Apc*^*Min/+*^ mice. **A** Quantification of F4/80^+^Arg1^+^ cells in the colon of 2% DSS treated *Apc*^*Min/+*^ mice with the indicated genotypes. **B** Quantification of F4/80^+^Nos2^+^ cells in the colon of 2% DSS treated *Apc*^*Min/+*^ mice with the indicated genotypes. **C** Quantification of MPO^+^Arg1^+^ cells in the colon of 2% DSS treated *Apc*^*Min/+*^ mice with the indicated genotypes. **D** Quantification of MPO^+^Nos2^+^ cells in the colon of 2% DSS treated *Apc*^*Min/+*^ mice with the indicated genotypes. **E** Quantification of CD3^+^CD8^+^ cells in the colon of 2% DSS treated *Apc*^*Min/+*^ mice with the indicated genotypes. **F** Quantification of CD3^+^CD4^+^ cells in the colon of 2% DSS treated *Apc*^*Min/+*^ mice with the indicated genotypes. **G** Quantification of CD8^+^GzmB^+^ cells in the colon of 2% DSS treated *Apc*^*Min/+*^ mice with the indicated genotypes. **H** Quantification of CD4^+^Foxp3^+^ cells in the colon of 2% DSS treated *Apc*^*Min/+*^ mice with the indicated genotypes. **I** Graphical summary of results and conclusions obtained in this study. **A**–**H** Three mice per genotype were evaluated. Mean values ± SEM are provided. Student’s *t* test was used to determine significance. *p < 0.05, **p < 0.01.

In order to determine whether myeloid *Mir34a*-deficiency affects the distribution of tumor-associated myeloid cells in DSS-induced colon cancers of *Apc*^*Min/+*^ mice, we next evaluated the number of F4/80- and MPO-positive cells. Interestingly, a different pattern than in 18-week-old mice without DSS-treatment was observed after treatment with 2% DSS, with higher numbers of F4/80^+^ macrophages, but a decrease in MPO^+^ neutrophiles in colon cancers from *Apc*^*Min/+*^*; Mir34a*^*ΔMye*^ mice when compared to *Apc*^*Min/+*^ mice, indicating that macrophages might play a more important role than neutrophils in colon cancer progression than in the formation of adenomas in the small intestine (Figs. 7J, K and S8C). Nevertheless, the number of Arg1^+^ cells was elevated, while the number of Nos2^+^ cells was reduced in colon cancers of *Mir34a*-deficient mice (Fig. S8C), which was also seen in the SI of 18-week-old mice (Fig. S2B). In support of a tumor suppressive effect of myeloid *Mir34a* in macrophages, we verified that there were significantly more F4/80^+^Arg1^+^ cells and fewer F4/80^+^Nos2^+^ cells in colon cancers of *Apc*^*Min/+*^*; Mir34a*^*ΔMye*^ mice (Figs. 8A, B and S9A, B). A similar pattern was observed in MPO^+^ neutrophils, with significantly more MPO^+^Arg1^+^ cells and fewer MPO^+^Nos2^+^ cells (Figs. 8C, D and S9C, D). To further determine whether myeloid *Mir34a* indeed affects T-cell populations and their functional state in DSS-induced colon cancers of *Apc*^*Min/+*^ mice, we evaluated the same markers as for the age-matched *Apc*^*Min/+*^ mice. We detected increased numbers of CD4^+^ T-cells and Foxp3^+^ T_regs_ in colon cancers from *Apc*^*Min/+*^*; Mir34a*^*ΔMye*^ mice when compared to *Apc*^*Min/+*^ control mice, while CD8^+^ T-cells and Granzyme-B^+^ cytotoxic T-cells were reduced (Figs. 8E–H and S10A–D).

Therefore, loss of myeloid *Mir34a* function shifts both TAMs and TANs to pro-tumorigenic phenotypes in an inflammatory microenvironment. This shift in turn leads to a more immunosuppressive environment, presumably by increasing the number of CD4^+^Foxp3^+^ T_reg_ cells. Overall, these findings show that myeloid *Mir34a* has a pivotal role in inhibiting macrophage infiltration into colon cancers and in suppressing pro-tumoral myeloid cell polarization in the TME of colon cancers induced by DSS in *Apc*^*Min/+*^ mice.

## Discussion

In this study, we showed that myeloid *Mir34a*-deficiency promotes adenoma initiation and progression in *Apc*^*Min/+*^ mice by facilitating an increased number and shift in the polarization of tumor-associated myeloid cells towards a pro-tumorigenic state. We show that *Mir34a*-deficient BMDMs enhance the migration of co-cultured CT-26 colorectal cancer cells. Therefore, *Mir34a*-deficient macrophages presumably secrete factors that affect the migratory capacities of cancer cells. A likely scenario is that *Mir34a*-proficient macrophages prevent EMT of cancer cells in a paracrine manner, whereas loss of *Mir34a* promotes EMT, which results in increased migration and presumably invasion. One unexpected consequence of myeloid *Mir34a* loss was the elevated frequency of initiation of micro-adenomas throughout the intestinal tract. Therefore, myeloid *Mir34a* acts at an early time-point to suppress adenoma formation driven by mutations in the APC/WNT pathway. As similar mutations initiate the development of sporadic CRCs [29], this function of *miR-34a* may also apply to sporadic CRCs. Furthermore, myeloid *Mir34a* also suppressed inflammation-induced, colonic tumorigenesis and progression resulting from treatment of *Apc*^*Min/+*^ mice with DSS.

It was shown, that p53 promotes a tumor-suppressive immune response in both tumor cell and myeloid cells [30–33]. The loss of *p53* in tumor cells has been shown to facilitate an immune-escape [30]. In another study, it was demonstrated that p53 activation in myeloid cells limits tumor development by preventing the polarization towards anti-inflammatory/pro-tumoral M2-like TAMs in *Apc*^*Min/+*^ mice [32]. Our findings imply that this effect is, at least in part, mediated through *Mir34a*, which is a direct transcriptional target of p53 [34], since loss of myeloid *Mir34a* promoted accumulation of M2-like TAMs and thereby presumably enhanced tumor invasion.

Several *Mir34a* target mRNAs, such as *Pd-l1* [35], *Csf1r* [19, 36], *Ccl22* [37, 38] and *MMP9* [39], were upregulated in neoplasms as well as in BMDMs obtained from myeloid *Mir34a*-deficient *Apc*^*Min/+*^ mice. Therefore, myeloid *Mir34a* presumably maintains TAMs in M1-like states by suppressing the translation and abundance of mRNAs related to myeloid cell polarization. We have previously shown, that Csf1r expression is increased on mRNA and protein levels in *Mir34a*-deficient BMDMs and that Csf1r inhibition or *Csf1r* deletion in *Mir34a*-deficient myeloid cells attenuates colitis-associated colon-cancer/CAC development [20]. Consistent with this, we found in this study, that *Csf1r* mRNA was significantly increased in 18-week-old, myeloid *Mir34a*-deficient tumors from *Apc*^*Min/+*^ mice. Therefore, *Mir34a*-mediated suppression of *Csf1r* expression may promote polarization towards an M1-like, tumor suppressive state of TAMs.

Elevated expression of matrix metalloproteinase-9 (MMP9), which is encoded by a known miR-34a target mRNA [39], was detected in colon cancers and is associated with disease progression and decreased survival [40]. Vinnakota et al. showed that myeloid effector cells, such as M2-like macrophages, activate MMP9, which promotes epithelial-mesenchymal transition (EMT) and enhances invasion of the SW480 colorectal cell-line [41]. Our results indicate that *Mir34a* limits MMP9 expression and thereby slows down tumor progression.

MiR-34a directly targets the *PD-L1* mRNA, thereby reducing PD-L1 expression and enhancing T-cell-mediated immune responses along with decreasing T-regulatory cells [35]. In our study, we observed an increase of CD4^+^Foxp3^+^ cells in *Apc*^*Min/+*^ mice with myeloid *Mir34a*-deficiency. Therefore, *Pd-l1*, which was up-regulated in *Mir34a*-deficient myeloid cells in this study, might act as a key downstream effector of myeloid *Mir34a*. Additionally, it was shown that macrophage-derived Ccl22 attracts CCR4^+^ T_reg_ cells [42, 43] and that Il-10 produced by M2 macrophages promotes the transition of naïve T-cells into Foxp3^+^ Tregs [9, 44, 45]. In our study both, *Ccl22* and *Il-10*, were up-regulated in *Mir34a-*deficient BMDMs suggesting that enhancing *Mir34a* expression in myeloid cells might represent a promising strategy to counteract immune escape and improve immunotherapy efficacy in colorectal cancer. Although the restoration of *Mir34a* has not yet been established, we have previously shown, that the activation of *miR-34a* expression by activation of *miR-34a*-inducing transcription factors is possible: the chemo-preventive drugs Curcumin and salicylate/Aspirin induced *miR-34a* expression in a p53-independent manner by activating NRF2 in CRC cell lines [46, 47].

The results of this study imply that *Mir34a* has an important tumor suppressive role by regulating the polarization and activity of TAMs. However, in the TME of CRCs and presumably other entities, *Mir34a* may be down-regulated in myeloid cells due to hypoxia or inflammatory signals, since *miR-34a* is directly suppressed by HIF1α under hypoxic conditions [48, 49], and STAT3 activation leads to direct repression of *miR-34a* [50]. Our results imply, that hypoxia, which commonly occurs in late-stage primary tumors, may favor M2-polarization of TAMs, at least in part, due to down-regulation of *miR-34a* and thereby facilitates tumor progression.

In this study, an increase in the number of MPO-positive cells was observed, along with enhanced expression of *Cxcr2*, *Cxcl1* and *Cxcl2*, which are involved in neutrophil recruitment [51], in both BMDMs and tumor tissue from myeloid *Mir34a*-deficient *Apc*^*Min/+*^ mice. In accordance, our previous study revealed enhanced neutrophil infiltration, which was presumably due to increased CXCR2 signaling in myeloid *Mir34a*-deficient colitis-associated colon cancers (CACs) [20]. Unexpectedly, the number of MPO^+^Nos2^+^ cells was increased and the number of MPO^+^Arg1^+^ cells was decreased in *Mir34a*-deficient *Apc*^*Min/+*^ mice, indicating an increased number of anti-tumoral neutrophils. However, in DSS-induced colon cancers of *Mir34a*-deficient *Apc*^*Min/+*^ mice the opposite results were observed. One explanation for these divergent results could be that TANs may be driven towards an anti-tumoral N1-like state in early stage of tumors, but towards a more immune-suppressive/pro-tumoral N2-like state later during tumor progression or under inflammatory conditions. Similar scenarios have been described previously [52–54]. Therefore, the effects of *Mir34a* in neutrophiles appear to be context-dependent.

Although this study shows that myeloid *Mir34a* plays an important role in preventing tumor progression, it still has some limitations which need to be addressed in the future. We showed that myeloid *Mir34a*-deficiency leads to a pro-tumorigenic, immuno-suppressive TME, which is mediating tumor progression and invasion. However, at this stage the exact mechanisms as to how myeloid *Mir34a*-deficiency drives this progression needs to be further investigated. Future studies should uncover how the mechanism underlying myeloid *Mir34a*-deficiency promotes tumor progression. A full understanding might also allow us identifying potential future therapy strategies in this scenario.

We found that loss of myeloid *Mir34a* facilitates the formation of invasive carcinomas in both the small intestine and the colon of *Apc*^*Min/+*^ mice resulting from enhanced polarization towards M2-like TAMs and N2-like TANs within tumors. Taken together, our findings demonstrate that myeloid *Mir34a* plays an important regulatory role within the TME by controlling myeloid cell plasticity, which ultimately prevents tumor progression.

## Supplementary information

Supplementary Material

## Data Availability

All data, analytic methods, and study materials will be made available to others.
