# Supplementary material for: Myeloid Mir34a suppresses initiation and progression of intestinal and colitis-induced colon cancers in APCmin mice
Source: Cell Death Dis. 2026 May 15;17(1):458. doi: 10.1038/s41419-026-08851-6 (PMC13179333; doi:10.1038/s41419-026-08851-6)
Supplement: Supplementary file 1 — Supplementary Material [file 41419_2026_8851_MOESM1_ESM.pdf]

# Supplementary Material

## Myeloid *Mir34a* suppresses initiation and progression of intestinal and colitis-induced colon cancers in *APC<sup>min</sup>* mice

Yun Chen, Fangteng Liu, Janine König, Nassim Bouznad and Heiko Hermeking

## List of contents

### Supplementary Tables

Table S1: Oligonucleotides

Table S2: Kits

Table S3: Antibodies

### Supplementary Figures

Supplementary Figure S1. Related to Figure 1.

Supplementary Figure S2. Related to Figure 2. and Figure 3.

Supplementary Figure S3. Related to Figure 3.

Supplementary Figure S4. Related to Figure 3.

Supplementary Figure S5. Related to Figure 4.

Supplementary Figure S6. Related to Figure 5.

Supplementary Figure S7. Related to Figure 6.

Supplementary Figure S8. Related to Figure 7.

Supplementary Figure S9. Related to Figure 8.

Supplementary Figure S10. Related to Figure 8.

## Supplementary Tables

**Table S1.** Oligonucleotides

| **Name** | **Sequence (5'-3')** | **Use** |
|---|---|---|
| *mmu-Cyclophilin* R | TTCTGCTGTCTTTGGAACTTTGTC | qPCR |
| *mmu-Cyclophilin* F | ATGGTCAACCCCACCGTGT | qPCR |
| *mmu-Arginase-1* F | GTGAAGACGGCAGTGGCT TT | qPCR |
| *mmu-Arginase-1* R | GTCCCTGGCTTATGGTTACCC | qPCR |
| *mmu-Nos2* F | GTTCTCAGCCCAACAATACAAGA | qPCR |
| *mmu-Nos2* R | GTGGACGGGTCGATGTCAC | qPCR |
| *mmu-Mrc1* F | TTGGTGGCAATTCACGAGAG | qPCR |
| *mmu-Mrc1* R | GGGAAGGGTCAGTCTGTGTTTG | qPCR |
| *mmu-Ym-1* F | CTGGAATTGGTGCCCCTACA | qPCR |
| *mmu-Ym-1* R | CAAGCATGGTGGTTTTACAGGA | qPCR |
| *mmu-Ccr2* F | ATC CAC GGC ATA CTA TCA ACA TC | qPCR |
| *mmu-Ccr2* R | TCGTAGTCATACGGTGTGGTG | qPCR |
| *mmu-Ccl2* F | CAGCCAGCAGTTAACGC | qPCR |
| *mmu-Ccl2* R | AGCCTACTCATTGGGATCATCTTG | qPCR |
| *mmu-Cxcl10* F | CCAAGTGCTGCCGTCATTTTC | qPCR |
| *mmu-Cxcl10* R | GGCTCGCAGGGATGATTTCAA | qPCR |
| *mmu-Cxcr3* F | GGTTAGTGAACGTCAAGTGCT | qPCR |
| *mmu-Cxcr3* R | CCCCATAATCGTAGGGAGAGG | qPCR |
| *mmu-Cxcr2* F | CTTTGCCCTGACCTTGCCT | qPCR |
| *mmu-Cxcr2* R | GCGGTCCATGCTGATGC | qPCR |
| *mmu-Cxcl1* F | TAT CGC CAA TGA GCT GCG | qPCR |
| *mmu-Cxcl1* R | GGATGTTCTTGAGGTGAATCCC | qPCR |
| *mmu-Cxcl2* F | CCAACCACCAGGCTACAGG | qPCR |
| *mmu-Cxcl2* R | GCGTCACACTCAAGCTCTG | qPCR |
| *mmu-Ccl22* F | AGGTCCCTATGGTGCCAATGT | qPCR |
| *mmu-Ccl22* R | CGGCAGGATTTTGAGGTCCA | qPCR |
| *mmu-Csf1r* F | TGTCATCGAGCCTAGTGGC | qPCR |
| *mmu-Csf1r* R | CGGGAGATTCAGGGTCCAAG | qPCR |
| *mmu-Pd-l1* F | GGAGAAATGTGGCGTTGAAG | qPCR |
| *mmu-Pd-l1* R | ATCGACGATCAGAGGGTTCA | qPCR |
| *mmu-Mmp9* F | CAGCTGGCAGAGGCATACTTG | qPCR |
| *mmu-Mmp9* R | GCTTCTCTCCCATCATCTGGG | qPCR |
| *mmu-c-Myc* F | TGACCTAACTCGAGGAGGAGCTGGAATC | qPCR |
| *mmu-c-Myc* R | AAGTTTGAGGCAGTTAAAATTATGGCTGAAGC | qPCR |
| *mmu-Il-4* F | ACAGGAGAAGGGACGCCAT | qPCR |
| *mmu-Il-4* R | GAAGCCCTACAGACGAGCTCA | qPCR |
| *mmu-Il-10* F | GGTTGCCAAGCCTTATCGGA | qPCR |
| *mmu-Il-10* R | ACCTGCTCCACTGCCTTGCT | qPCR |
| *mmu-pri-Mir34a F* | CTGTGCCCTCTTGCAAAAGG | qPCR |
| *mmu-pri-Mir34a R* | GGACATTCAGGTGAGGGTCTTG | qPCR |
| *mmu-370* (Cre) Fwd | ACCTGAAGATGTTCGCGATTATCT | GT |
| *mmu-370* (Cre) Rev | ACCGTCAGTACGTGAGATATCTT | GT |
| *mmu-144* (Cre) Fwd | CACAGTGCCCACATTATTTAGATA | GT |
| *mmu-321* (Cre) Rev | GTCTTCAACCTCCCAAGCCTT | GT |
| *mmu-Mir34a* FWD | ACCTTGCAGGTGCTCAGAAT | GT |
| *mmu-Mir34a* R2 | TGGAGCTAACGGAGTGTGTG | GT |
| *mmu-Mir34a* R4 | CTACCCAAGCTCGACGAAGT | GT |
| *mmu-Mir34a* R8 | TGCAGCACTTCTAGGGCAGT | GT |
| *mmu- APCmin-Mut* | TTCTGAGAAAGACAGAAGTTA | GT |
| *mmu- APCmin-Common* | TTCCACTTTGGCATAAGGC | GT |
| *mmu- APCmin-Wt* | GCCATCCCTTCACGTTAG | GT |

qPCR: Quantitative real-time PCR; GT: Genotyping

**Table S2.** Kits

| Name | Catalog Number | Company | Use |
|---|---|---|---|
| Impress®HRP Horse Anti-Rabbit IgG Polymer Kit | MP-7401 | Vector laboratories Inc. | IHC |
| M.O.M. ® (Mouse on Mouse) Immunodetection Kit, Basic | BMK-2202 | Vector laboratories Inc. | IHC |
| VECTASTAIN®Elite® ABC Universal Kit, Peroxidase, R.T.U (Horse Anti-Mouse/Rabbit IgG) | PK-7200 | Vector laboratories Inc. | IHC |
| Verso cDNA Synthesis Kit | AB-1453/B | Thermo Fisher Scientific | PCR |
| miRCURY® LNA® RT Kit | 339340 | Qiagen | PCR |
| miRNeasy® Mini Kit | 74104 | Qiagen | PCR |
| miRCURY® LNA® SYBR® Green PCR Kit | 339346 | Qiagen | PCR |
| mature miR-34a | YP00204486 | Qiagen | PCR |
| SNORD48 | YP00203903 | Qiagen | PCR |

IHC: Immunohistochemistry

**Table S3.** Antibodies

| Name | Species | Catalog Number | Company | Use | Dilution | Source |
|---|---|---|---|---|---|---|
| F4/80 | Mouse/rat | 29414-1-AP | Proteintech | IHC<br>IF | 1:6000<br>1:500 | rabbit |
| Myeloperoxidase (MPO) | human | A0398 | Agilent Technologies | IHC<br>IF | 1:600<br>1:300 | rabbit |
| Arginase-1 | mouse/rat | sc-271430 | Santa Cruz Biotechnology | IHC<br>IF | 1:200<br>1:200 | mouse |
| Nos2 (iNOS) | mouse/rat /human | sc-7271 | Santa Cruz Biotechnology | IHC<br>IF | 1:50<br>1:400 | mouse |
| CD3 | mouse/human | A0452 | DAKO | IF | 1:150 | rabbit |
| CD4 | mouse | 14-9766-82 | Invitrogen | IF | 1:100 | rat |
| CD8a | mouse | 14-0808-82 | Invitrogen | IF | 1:100 | rat |
| FoxP3 | mouse/monkey | 12653 | Cell signaling | IF | 1:100 | rabbit |
| Granzyme-B | mouse/human | 17215 | Cell signaling | IF | 1:600 | rabbit |
| Cleaved-caspase 3 | mouse | 9661S | Cell signaling | IHC | 1:200 | rabbit |
| Ki-67 | mouse | 12202S | Cell signaling | IHC | 1:400 | rabbit |
| AF555 | goat | 2090527 | Invitrogen | IF | 1:500 | mouse |
| AF647 | goat | A32733 | Invitrogen | IF | 1:500 | rabbit |
| AF555 | goat | A21434 | Invitrogen | IF | 1:500 | rat |

IHC: Immunohistochemistry; IF: indirect immunofluorescence analysis

## Supplementary Figures

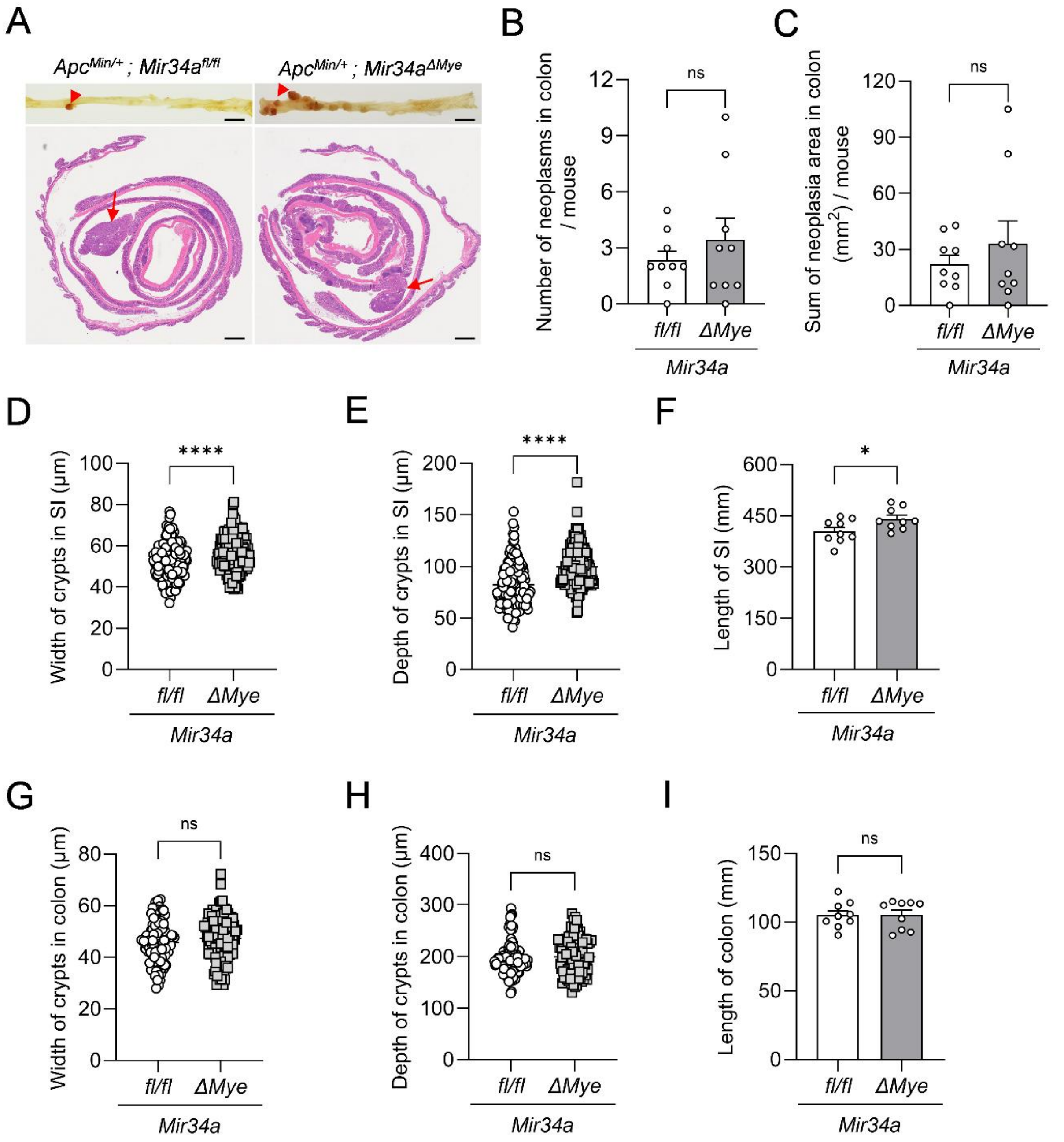


**Figure S1. (A)** Representative macroscopic images of polyps in resected colon of mice with the indicated genotypes. Scale bar: 2 cm. Representative H&E stained "swiss-roll" sections of the colon of 18-week-old *Apc*$^{Min/+}$ mice. Scale bar: 800 µm. **(B)** Quantification of the number of neoplasms in colon per mouse of 18-week-old *Apc*$^{Min/+}$ mice with the indicated genotypes (n=9 per genotype). **(C)** Sum of neoplasia area of colon per mouse of *Apc*$^{Min/+}$ mice with the indicated genotypes (n=9 per genotype). **(D)** Width and **(E)** depth of crypts in small intestine of 18-week-old *Apc*$^{Min/+}$ mice with indicated genotypes (n=9 per genotype). SI, small intestine. **(F)** Length of small intestine in mice with indicated genotypes (n=9 per genotype). **(G)** Width and **(H)** depth of crypts in colon of mice with indicated genotypes (n=9 per genotype). **(I)** Length of colon in mice with indicated genotypes (n=9 per genotype). **(B-I)** Mean values ±SEM are provided. Students t-test was used to determine significance. *$P$ < 0.05, ****$P$ < 0.0001, ns, not significant.

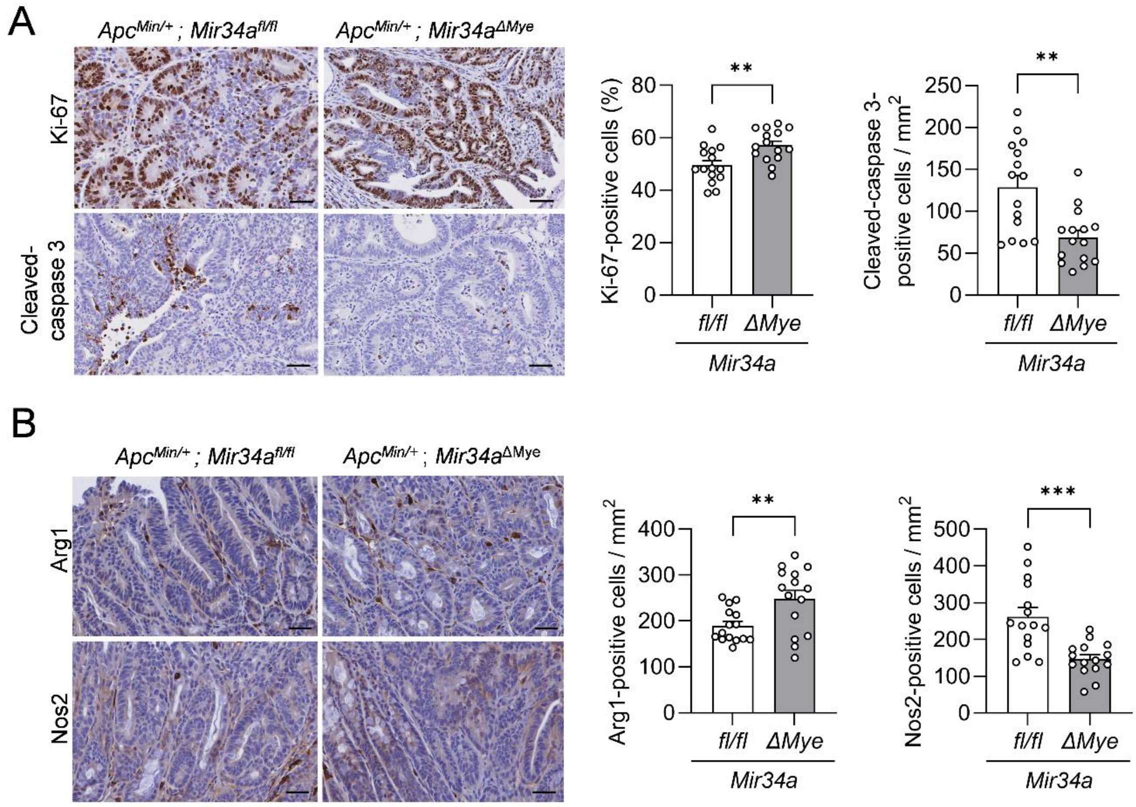


**Figure S2. (A)** Immunohistochemistry of Ki-67 and cleaved-caspase 3 in neoplasms from the colon of 18-weeks-old *Apc*$^{Min/+}$ mice with the indicated genotypes (left panel). Scale bars: 40 µm. Quantification of Ki-67 and cleaved-caspase 3 (right panel; n=3 per genotype). **(B)** Immunohistochemistry of Arg1 and Nos2 in neoplasms from the small intestine of 18-weeks-old *Apc*$^{Min/+}$ mice with the indicated genotypes (left panel). Scale bars: 40 µm. Quantification of Arg1 and Nos2 (right panel; n=3 per genotype). **(A-B)** Mean values ±SEM are provided. Students t-test was used to determine significance. $^{**}P < 0.01$, $^{***}P < 0.001$.

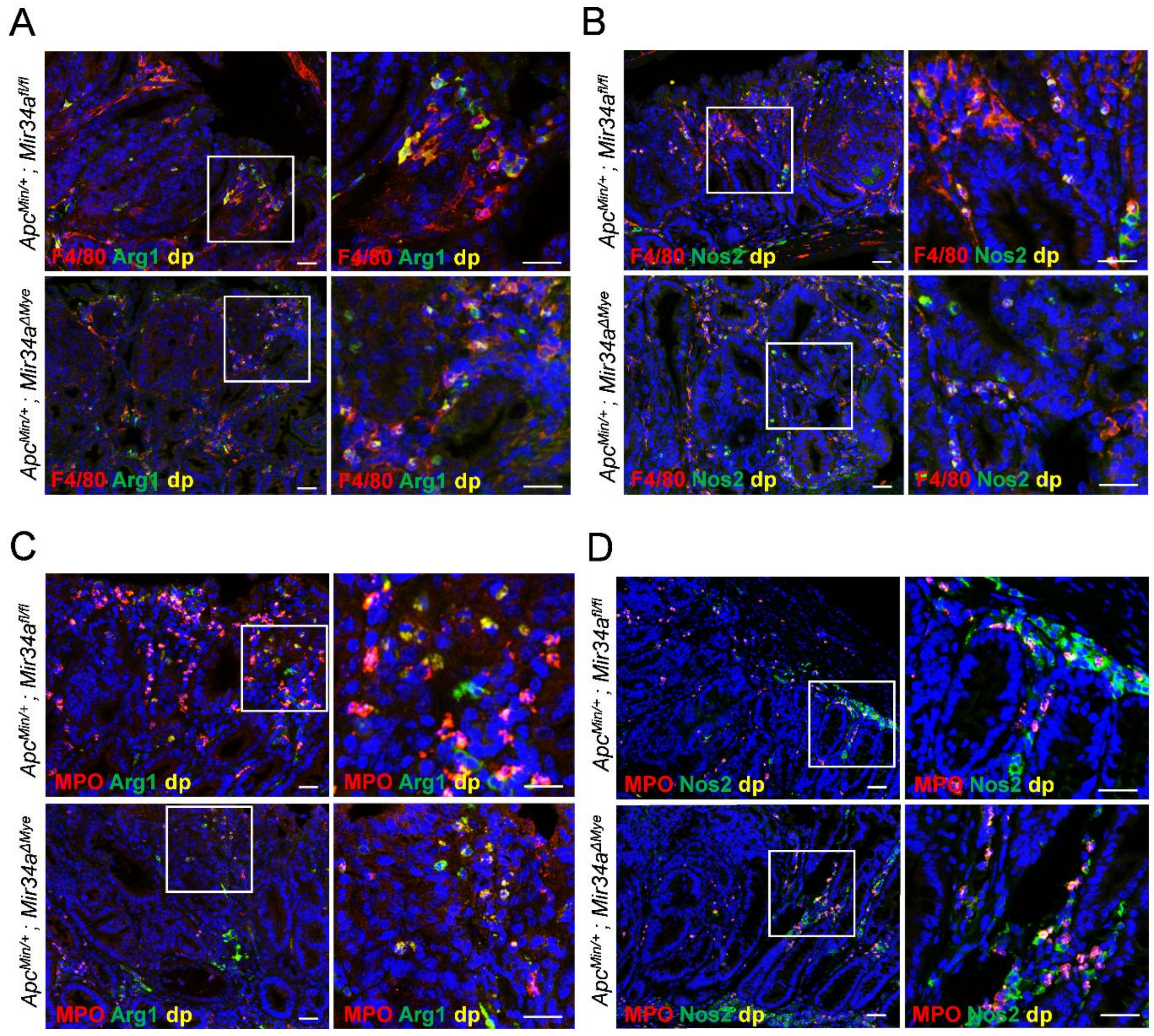


**Figure S3. (A-B)** Representative pictures of F4/80⁺Arg1⁺ and F4/80⁺Nos2⁺ macrophages in the small intestine of 18-week-old *Apc*$^{Min/+}$ mice with the indicated genotypes (left panel). Scale bar: 50 µm. Right panel corresponds to the indicated area in left panel. Scale bar: 20 µm. F4/80 (red), Arg1 (green), Nos2 (green), DAPI (blue) and dp (double positive, yellow). **(C-D)** Representative pictures of MPO⁺Arg1⁺ and MPO⁺Nos2⁺ neutrophils in the small intestine of 18-week-old *Apc*$^{Min/+}$ mice with the indicated genotypes (left panel). Scale bar: 50 µm. Right panel corresponds to the indicated area in left panel. Scale bar: 20 µm. MPO (red), Arg1 (green), Nos2 (green), DAPI (blue) and dp (double positive, yellow).

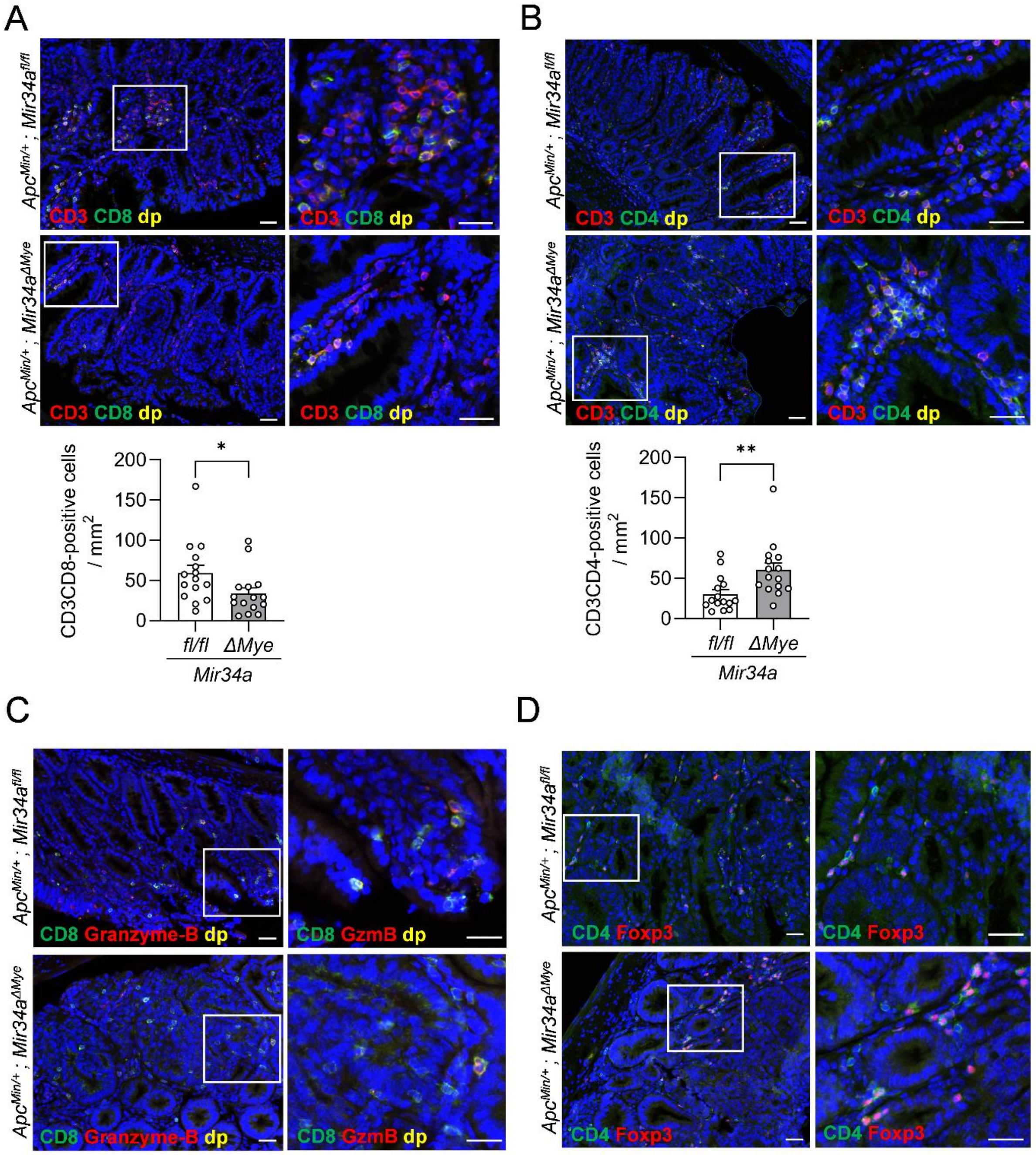


**Figure S4. (A)** Representative pictures of CD3⁺CD8⁺ T-cells in the small intestine of 18-week-old *Apc*$^{Min/+}$ mice with the indicated genotypes (left panel). Scale bar: 50 µm. And their boxed area in left panel (right panel). Scale bar: 20 µm. CD3 (red), CD8 (green), DAPI (blue) and dp (double positive, yellow). Quantification of $CD3^{+}CD8^{+}$ cells. **(B)** Representative pictures of $CD3^{+}CD4^{+}$ T-cells in the small intestine of 18-week-old *Apc*$^{Min/+}$ mice with the indicated genotypes (left panel). Scale bar: 50 µm. And their boxed area in left panel (right panel). Scale bar: 20 µm. CD3 (red), CD4 (green), DAPI (blue) and dp (double positive, yellow). Quantification of $CD3^{+}CD4^{+}$ cells. **(C)** Representative pictures of co-stained Granzyme-B positive cytotoxic T-cells in the small intestine of 18-week-old *Apc*$^{Min/+}$ mice with the indicated genotypes (left panel). Scale bar: 50 µm. Right panel corresponds to the indicated area in left panel. And their boxed area in left panel (right panel). Scale bar: 20 µm. Granzyme-B (red), CD8 (green), DAPI (blue) and dp (double positive, yellow). **(D)** Representative pictures of co-stained Foxp3 positive regulatory T-cells in the small intestine of 18-week-old *Apc*$^{Min/+}$ mice with the indicated genotypes (left panel). Scale bar: 50 µm. Right panel corresponds to the indicated area in left panel. Scale bar: 20 µm. Foxp3 (red), CD4 (green), and DAPI (blue). **(A-B)** Three mice per genotype were evaluated. Mean values ±SEM are provided. Students t-test was used to determine significance. *$P < 0.05$, **$P < 0.01$.

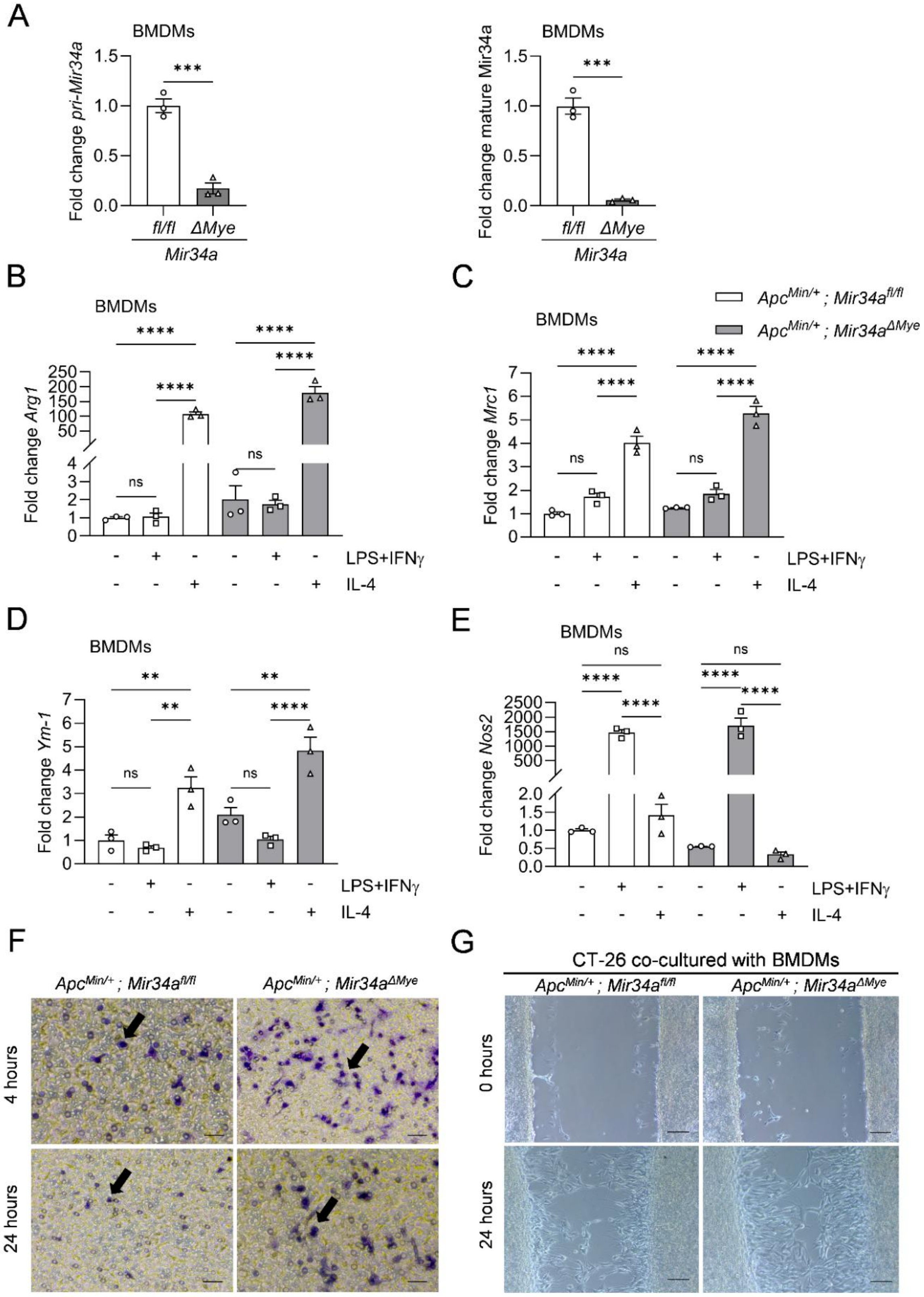


**Figure S5.** **(A)** qPCR analysis of *pri-Mir34a* expression (left panel) and mature Mir34a expression (right panel) in BMDMs of *Apc*$^{Min/+}$ mice with the indicated genotypes (n=3 per genotype). **(B-E)** Expression of **(B)** Arg1, **(C)** Mrc1, **(D)** Ym-1, and **(E)** Nos2 in BMDMs of *Apc*$^{Min/+}$ mice with the indicated genotypes after treatment with IL-4 or LPS + IFN-γ for 4 hours (n=3 per genotype). **(F)** Modified Boyden chamber assay was performed in triplicate inserts. Crystal violet staining of BMDMs, which migrated through 8 µm pores. Arrows indicate BMDMs. Scale bars: 20 µm. **(G)** Wound closure of CT-26 cells co-cultured with *Mir34a*-proficient or *Mir34a*-deficient BMDMs from *Apc*$^{Min/+}$ mice was performed in triplicate inserts for 24 hours. Scale bars: 50 µm. **(A-E)** Mean values ±SEM are provided. Students t-test was used to determine significance. $^{**}P < 0.01$, $^{***}P < 0.001$, $^{****}P < 0.0001$, ns, not significant.

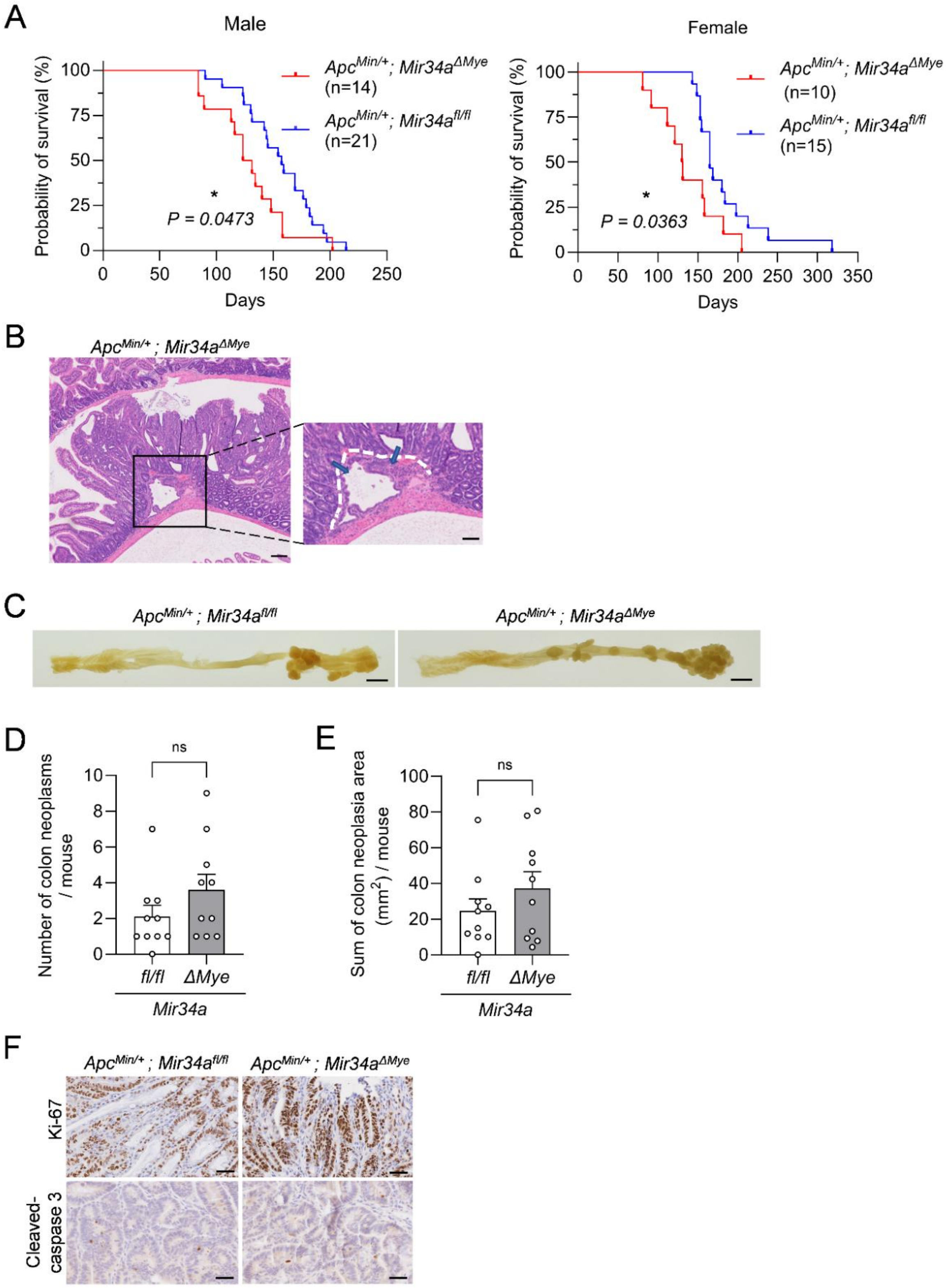


**Figure S6. (A)** Kaplan-Meier survival analysis of male (left) and female (right) $Apc^{Min/+}$ mice with the indicated genotypes. Results were compared with a log-rank test. **(B)** Representative picture of an invasive area in the small intestine of $Apc^{Min/+}$; $Mir34a^{\Delta Mye}$ mice. Scale bars: 800µm (left); 300µm (right). **(C)** Representative macroscopic images of polyps in resected colon of 18-week-old $Apc^{Min/+}$ mice with the indicated genotypes. Scale bars: 1cm. **(D)** Quantification of the number of neoplasms in the colon per mouse of 18-week-old $Apc^{Min/+}$ mice with the indicated genotypes (n=10 per genotype). **(E)** Sum of neoplasia area in the colon per mouse of $Apc^{Min/+}$ mice with the indicated genotypes (n=10 per genotype). **(F)** Immunohistochemistry of Ki-67 and cleaved-caspase 3 in neoplasms from the small intestine in moribund $Apc^{Min/+}$ mice with the indicated genotypes. Scale bars: 40 µm. **(D, E)** Mean values ±SEM are provided. Students t-test was used to determine significance. ns, not significant.

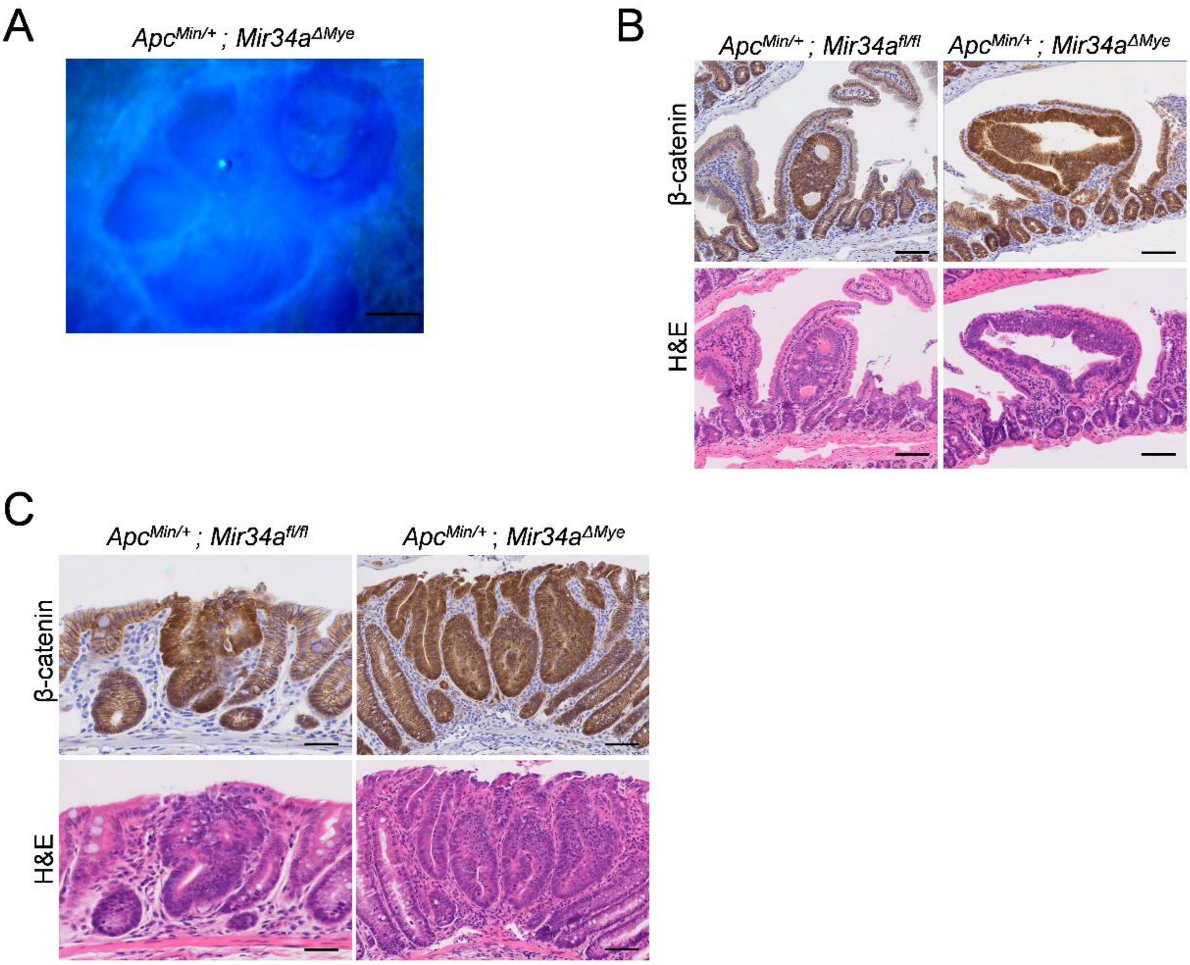


**Figure S7. (A)** Microscopic picture of methylene blue stained AFC. Scale bar: 5× magnification. **(B)** Representative pictures of β-catenin and H&E staining of micro-adenomas in the small intestine of 5-week-old *Apc*$^{Min/+}$ mice with indicated genotypes. Scale bars: 50µm. **(C)** Representative pictures of β-catenin and H&E staining micro-adenomas in the colon of 5-week-old *Apc*$^{Min/+}$ mice with indicated genotypes. Scale bars: 50µm.

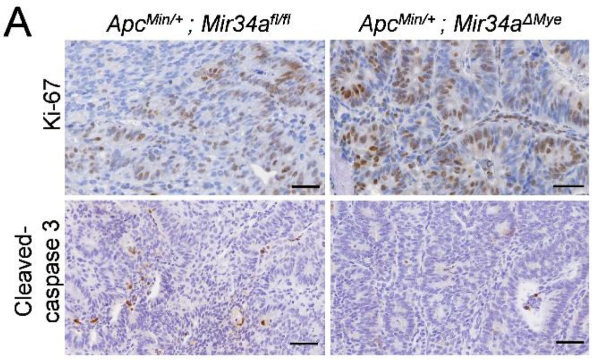


B

| | Classification | | | |
|---|---|---|---|---|
| | Adenomas | | Carcinomas | |
| | Low-grade dysplasia | High-grade dysplasia | Invasive | Total number of neoplasms |
| *Apc*$^{Min/+}$ ; *Mir34a*$^{fl/fl}$ | 25 (50.00%) | 25 (50.00%) | 0 (0.0%) | 50 |
| *Apc*$^{Min/+}$ ; *Mir34a*$^{\Delta Mye}$ | 36 (49.32%) | 31 (42.47%) | 6 (8.22%) | 73 |

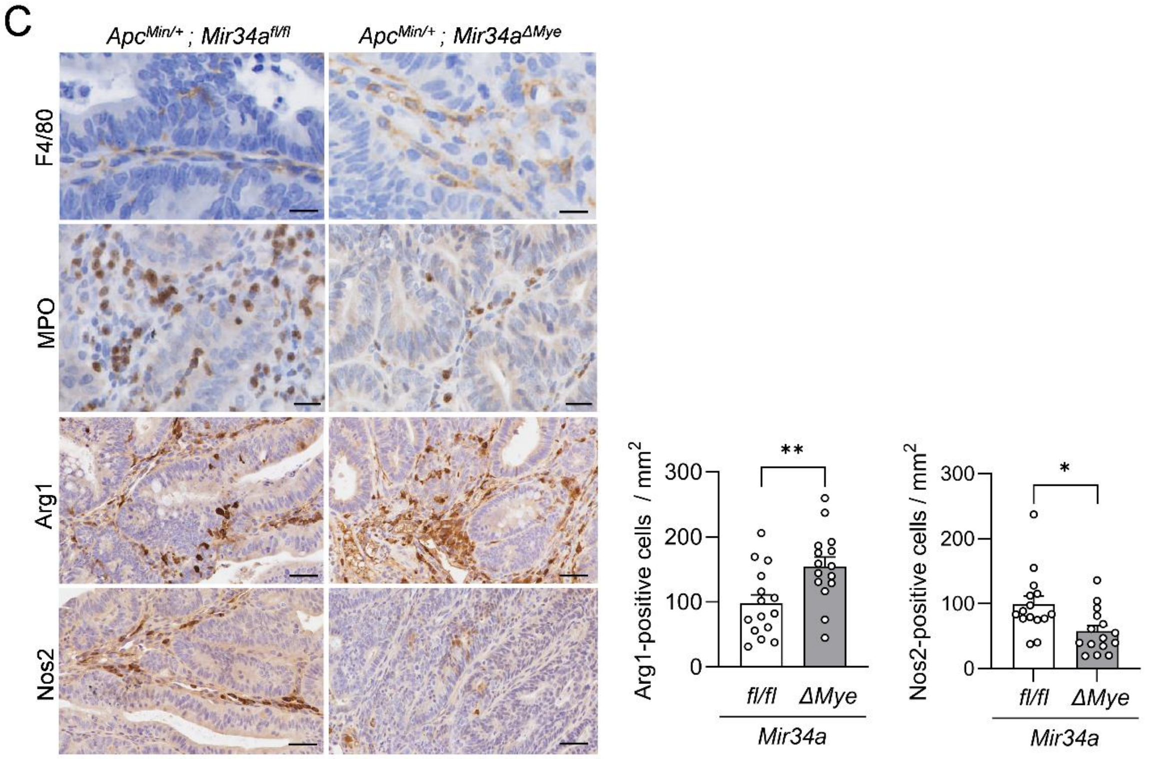


**Figure S8. (A)** Detection of Ki-67 and cleaved-caspase 3 by Immunohistochemistry in neoplasms from the colon in 2% DSS treated *Apc*$^{Min/+}$ mice with the indicated genotypes. Scale bars: 40 µm. **(B)** Quantification of different tumor stages and tumor number in tumors from the colon in 2% DSS treated *Apc*$^{Min/+}$ mice with the indicated genotypes (n=4 per genotype). **(C)** Immunohistochemistry of F4/80, MPO, Arg1 and Nos2 in neoplasms from the colon in 2% DSS treated *Apc*$^{Min/+}$ mice with the indicated genotypes (left panel). Scale bars: 40 µm. Quantification of Arg1 and Nos2 (right panel, n=3 per genotype). Mean values ±SEM are provided. Students t-test was used to determine significance. *$P$ < 0.05, **$P$ < 0.01.

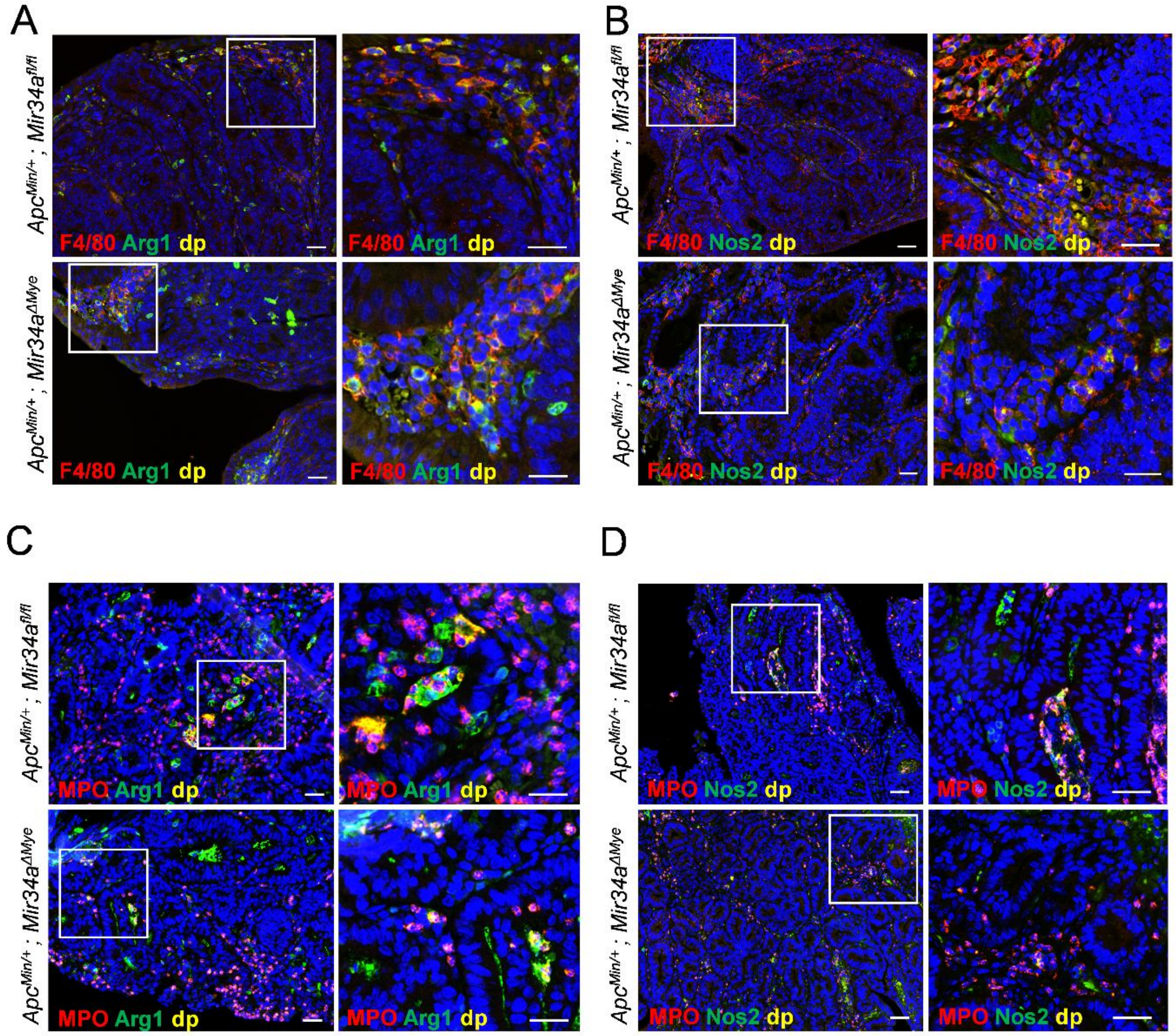


**Figure S9. (A)** Representative pictures of F4/80$^+$Arg1$^+$ macrophages in the colon of 2% DSS treated *Apc$^{Min/+}$* mice with the indicated genotypes (left panel). Scale bar: 50 µm. And their boxed area in left panel (right panel). Scale bar: 20 µm. F4/80 (red), Arg1 (green), DAPI (blue) and dp (double positive, yellow). **(B)** Representative pictures of F4/80$^+$Nos2$^+$ macrophages in the colon of 2% DSS treated *Apc$^{Min/+}$* mice with the indicated genotypes (left panel). Scale bar: 50 µm. And their boxed area in left panel (right panel). Scale bar: 20 µm. F4/80 (red), Nos2 (green), DAPI (blue) and dp (double positive, yellow). **(C)** Representative pictures of MPO$^+$Arg1$^+$ neutrophils in the colon of 2% DSS treated *Apc$^{Min/+}$* mice with the indicated genotypes (left panel). Scale bar: 50 µm. And their boxed area in left panel (right panel). Scale bar: 20 µm. MPO (red), Arg1 (green), DAPI (blue) and dp (double positive, yellow). **(D)** Representative pictures of MPO$^+$Nos2$^+$ neutrophils in the colon of 2% DSS treated *Apc$^{Min/+}$* mice with the indicated genotypes (left panel). Scale bar: 50 µm. And their boxed area in left panel (right panel). Scale bar: 20 µm. MPO (red), Nos2 (green), DAPI (blue) and dp (double positive, yellow).

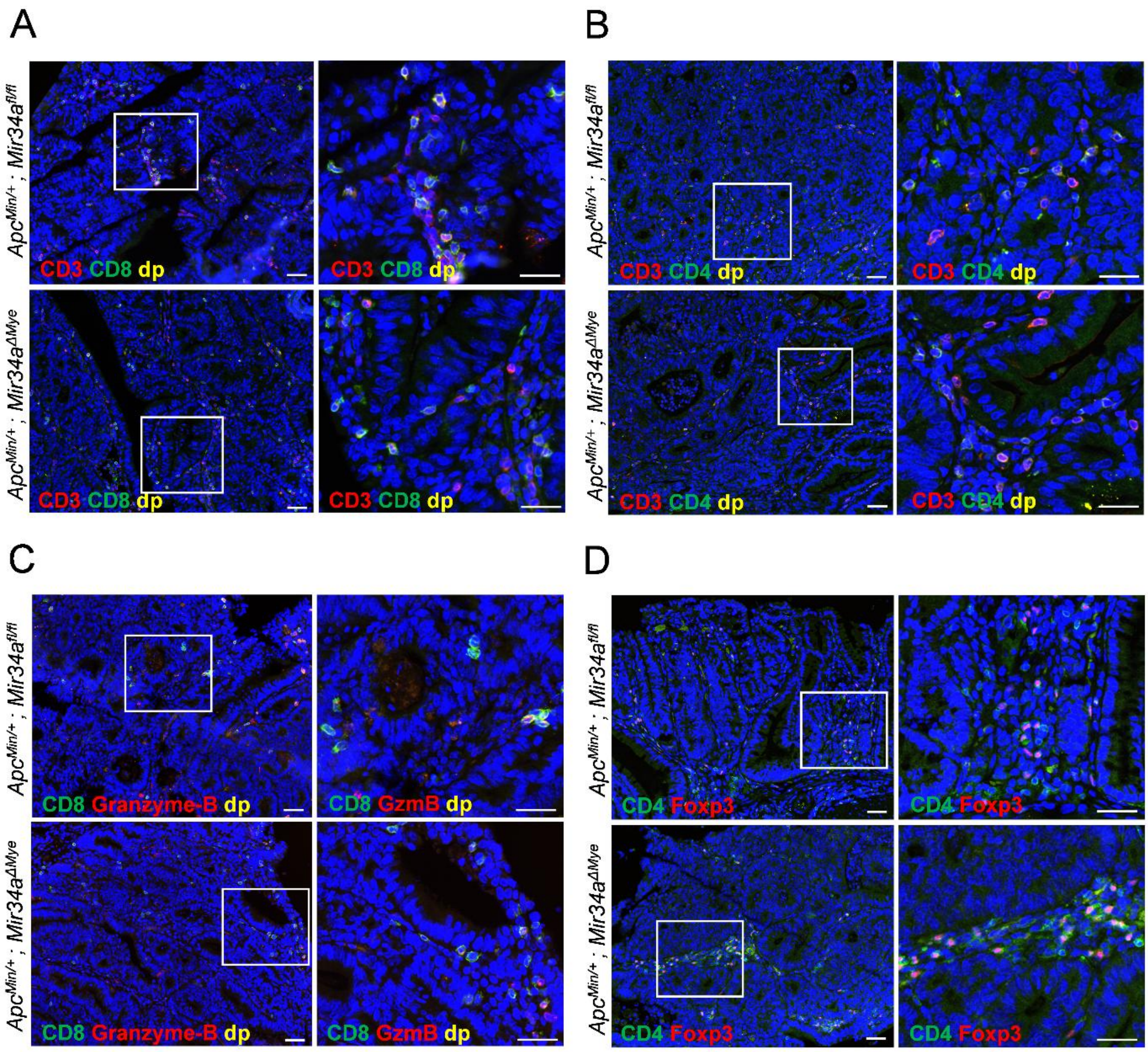


**Figure S10. (A)** Representative pictures of $CD3^+CD8^+$ T-cells in the colon of 2% DSS treated *Apc*$^{Min/+}$ mice with the indicated genotypes (left panel). Scale bar: 50 µm. And their boxed area in left panel (right panel). Scale bar: 20 µm. CD3 (red), CD8 (green), DAPI (blue) and dp (double positive, yellow). **(B)** Representative pictures of $CD3^+CD4^+$ T-cells in the colon of 2% DSS treated *Apc*$^{Min/+}$ mice with the indicated genotypes (left panel). Scale bar: 50 µm. And their boxed area in left panel (right panel). Scale bar: 20 µm. CD3 (red), CD4 (green), DAPI (blue) and dp (double positive, yellow). **(C)** Representative pictures of $CD8^+GzmB^+$ cytotoxic T-cells in the colon of 2% DSS treated *Apc*$^{Min/+}$ mice with the indicated genotypes (left panel). Scale bar: 50 µm. And their boxed area in left panel (right panel). Scale bar: 20 µm. Granzyme-B (red), CD8 (green), DAPI (blue) and dp (double positive, yellow). **(D)** Representative pictures of $CD4^+Foxp3^+$ regulatory T-cells in the colon of 2% DSS treated *Apc*$^{Min/+}$ mice with the indicated genotypes (left panel). Scale bar: 50 µm. And their boxed area in left panel (right panel). Scale bar: 20 µm. Foxp3 (red), CD4 (green), and DAPI (blue).
